# Elongator complex differentially regulates transcription and translation in the hypocotyl and cotyledons during early light-dependent *Arabidopsis* development

**DOI:** 10.1093/pcp/pcag005

**Published:** 2026-01-16

**Authors:** Magdalena Jarosz-Ostrówka, Małgorzata Kwaśniak-Owczarek, Natalia Małecka, Magda Mielczarek, Paweł Migdał, Olimpia Gagliardi, Mieke Van Lijsebettens, Paweł Mackiewicz, Magdalena Wołoszyńska

**Affiliations:** Department of Genetics, Faculty of Biology and Animal Science, Wrocław University of Environmental and Life Sciences, Kożuchowska 7, 51-631 Wrocław, Poland; Department of Bioinformatics and Genomics, Faculty of Biotechnology, University of Wrocław, F. Joliot-Curie 14a, Wrocław 50-383, Poland; Department of Cellular Molecular Biology, Faculty of Biotechnology, University of Wrocław, F. Joliot-Curie 14a, Wrocław 50-383, Poland; Department of Genetics, Faculty of Biology and Animal Science, Wrocław University of Environmental and Life Sciences, Kożuchowska 7, 51-631 Wrocław, Poland; Department of Genetics, Faculty of Biology and Animal Science, Wrocław University of Environmental and Life Sciences, Kożuchowska 7, 51-631 Wrocław, Poland; Department of Environment Hygiene and Animal Welfare, Faculty of Biology and Animal Science, Wrocław University of Environmental and Life Sciences, Chełmońskiego 38E, Wroclaw 51-630, Poland; VIB Center for Plant Systems Biology, Technologiepark 71, Ghent 9052, Belgium; Department of Biology, Ecology and Earth Sciences, University of Calabria, 87036 Arcavacata di Rende (CS), Italy; VIB Center for Plant Systems Biology, Technologiepark 71, Ghent 9052, Belgium; Department of Plant Biotechnology and Bioinformatics, Ghent University, Ghent 9052, Belgium; Department of Bioinformatics and Genomics, Faculty of Biotechnology, University of Wrocław, F. Joliot-Curie 14a, Wrocław 50-383, Poland; Department of Genetics, Faculty of Biology and Animal Science, Wrocław University of Environmental and Life Sciences, Kożuchowska 7, 51-631 Wrocław, Poland; Department of Cellular Molecular Biology, Faculty of Biotechnology, University of Wrocław, F. Joliot-Curie 14a, Wrocław 50-383, Poland

**Keywords:** *Arabidopsis thaliana*; Elongator complex, photomorphogenesis, seedling development, transcription, translation

## Abstract

During *Arabidopsis thaliana* photomorphogenesis, light promotes cotyledon expansion and inhibits hypocotyl elongation. This process involves transcriptional reprogramming controlled by various factors, including the Elongator complex, which regulates gene expression at the level of transcription and translation via epigenetic and transfer RNA modifications, respectively. The *elo3–6* mutant, lacking Elongator activity, exhibited photomorphogenic defects: less open, hyponastic cotyledons and an elongated hypocotyl. RNA-Seq and miRNA-Seq revealed distinct dysregulated gene sets in the *elo3–6* mutant hypocotyl and cotyledons. In the hypocotyl, the *elo3–6* defect affected the expression of genes involved in chloroplast physiology, circadian regulation, and auxin responses. Impaired chloroplast biogenesis apparently triggered retrograde signaling and a hypoxia-like state, preventing full inhibition of hypocotyl elongation. The defective *elo3–6* cotyledon development is likely due to compromised translation. This was supported by the presence of similar morphological defects in *urm11 urm12*, defective in the same type of tRNA modification as *elo3–6*, and the synergism observed in the *elo3–6 urm11 urm12* (*euu*) triple mutant showing seedling lethality. Moreover, *elo3–6* and *urm11 urm12* showed increased tolerance to translation inhibitors, including hygromycin B, which prevented the narrower cotyledon opening in *elo3–6*, suggesting that strong Elongator-dependent codon–anticodon interactions are required for proper cotyledon development. Interestingly, the genes enriched in codons recognized by tRNA anticodons modified by Elongator showed decreased mRNA abundance in *elo3–6*, suggesting a feedback mechanism downregulating the abundance of inefficiently translated mRNAs. Our results suggest that Elongator’s transcriptional role is more important in hypocotyl growth, while its translational role is more prominent in cotyledon development.

## Introduction

Being sessile, plants strongly depend on environmental factors, with light playing the pivotal role in their life cycle. In young seedlings, light triggers organ-specific transcriptome reprogramming leading to morphological and physiological changes. Seedlings germinating in darkness undergo skotomorphogenesis, characterized by closed cotyledons and elongated hypocotyl. Light exposure initiates photomorphogenesis, inducing distinct developmental changes in hypocotyl, which cease to elongate, and in cotyledons, which open and enlarge (Montgomery 2016). These two morphogenetic pathways are driven by opposing cell proliferation and elongation patterns in these organs. In darkness, cell expansion is promoted in the hypocotyl and inhibited in cotyledons. Upon light exposure, this pattern reverses, which is underpinned by profound transcriptional reprogramming (Ma et al. 2005), highlighting organ-specific gene expression in response to light (Kohnen et al. 2016, Sun et al. 2016, Dong et al. 2019). Cotyledons, the primary photosynthetic organs in the seedling, exhibit a pronounced light-induced upregulation of genes associated with chloroplast development and photosynthesis (Martín and Duque 2021). Although the hypocotyl is also photosynthetically active, its chloroplasts have higher oxygen evolution rates and show a greater capacity for the synthesis of photoprotective zeaxanthin (Jin et al. 2001). The transition from skoto- to photomorphogenesis is regulated by phytochrome-associated transcription factors. Positive regulators like ELONGATED HYPOCOTYL 5 (HY5) and its homolog HYH promote photomorphogenesis by repressing hypocotyl elongation (Oyama et al. 1997, Holm et al. 2002). In contrast, phytochrome interacting factors (PIFs) accumulate in darkness to maintain skotomorphogenesis. Light triggers their degradation, whereas HY5 and HYH are degraded in darkness (Kim et al. 2017, Paik et al. 2017). The interplay between the positive and negative factors ensures proper seedling morphogenesis in response to light.

Compared with the well-established transcriptional regulation of photomorphogenesis, its translational control remains less understood. mRNA translation is modulated by light and can be enhanced upon light exposure independently of respective transcript levels (Juntawong and Bailey-Serres 2012). Expression of numerous genes activated during photomorphogenesis is regulated at both the transcriptional and translational levels (Liu et al. 2013, Jang et al. 2019). However, some genes are regulated preferentially or exclusively at the translational level, with their transcript abundance remaining largely unchanged during the transition from darkness to light (Juntawong and Bailey-Serres 2012). The efficient translation of such mRNAs at the onset of photomorphogenesis is made possible by their release from cytoplasmic processing bodies that sequester the transcripts during skotomorphogenesis (Jang et al. 2019).

The Elongator complex, composed of six subunits, is essential for normal plant development. Plants mutated in genes encoding any of its subunits, known as *elo* mutants, exhibit defective growth during both skoto- and photomorphogenesis (Wołoszyńska et al. 2018). Elongator was initially identified in yeast (Otero et al. 1999), and then isolated from *Arabidopsis thaliana* (Nelissen et al. 2005). Its structural organization is conserved in eukaryotes, with the enzymatic activity residing in the ELP3 subunit, which harbors an N-terminal radical *S*-adenosylmethionine (SAM) domain and a C-terminal lysine (K) acetyltransferase (KAT) domain (Glatt and Müller 2013). In plants, the complex is found in both the cytoplasm and the cell nucleus (Nelissen et al. 2010). The cytoplasmic function of Elongator is related to its role in the fine-tuning of mRNA translation through modification of the 34th wobble uridine in the anticodon loop of specific tRNAs, first demonstrated in yeast (Durant et al. 2005, Esberg et al. 2006, Begley et al. 2007, Johansson et al. 2008). Later, it was found to be required for endoreduplication cycling during leaf development and proper auxin responses in plants (Mehlgarten et al. 2010, Leitner et al. 2015, Nakai et al. 2019, Jun et al. 2022). During the tRNA modification, the N-terminal domain of ELP3 binds and cleaves SAM, leading to the attachment of carboxymethyl group to the fifth position of the wobble uridine, which results in forming 5-carboxymethyluridine—cm^5^U_34_ (Huang et al. 2005). Following this modification, the uridine can be converted into 5-carbamoylmethyluridine (ncm^5^U_34_), 5-methoxycarbonylmethyluridine (mcm^5^), or 5-methoxycarbonylmethyl-2-thiouridine (mcm^5^s^2^U_34_). The thiolation is catalyzed by different enzymes, including ubiquitin-related protein modifier 1 (URM1)-like proteins URM11 and 12. ELP3-deficient plants lack the mcm and sulfur modification of the tRNA wobble uridine (Mehlgarten et al. 2010, Leitner et al. 2015). Modifications around the anticodon loop promote noncanonical base pairing with the mRNA codon in the ribosomal A-(aminoacyl) site, thereby contributing to the decoding accuracy and facilitating optimal translation speed (Nedialkova and Leidel 2015, Ranjan and Rodnina 2017). In yeast, Elongator modifies 11 tRNA species (Huang et al. 2005, Lu et al. 2005, Johansson et al. 2008), including three tRNAs that are additionally thiolated: tRNA^Glu^_UUC_, tRNA^Gln^_UUG_, and tRNA^Lys^_UUU_. In *Arabidopsis*, three tRNAs bearing the mcm modification and thiolation are targeted by Elongator (Mehlgarten et al. 2010, Leitner et al. 2015).

The nuclear function of Elongator is related to its role in DNA replication and transcription (Nelissen et al. 2010, Sanmartín et al. 2011, Antosz et al. 2017, Qi et al. 2019). In view of the enzymatic domains of ELP3 and experimental evidence showing altered epigenetic marks on genes downregulated in *elo* mutants, plant Elongator is believed to regulate gene expression through modulation of DNA methylation and/or acetylation of histones in the coding sequences, which activates the elongation phase of transcription. In *elo* mutants, altered cytosine methylation has been observed in genes associated with plant defense and cell cycle, and moderately lowered histone acetylation in genes implicated in growth promotion, root development, defense, circadian rhythm, and photomorphogenesis (Wang et al. 2013, Jia et al. 2015, Wang et al. 2015, An et al. 2017, Wołoszyńska et al. 2018, Qi et al. 2019, Jarosz et al. 2020). Auxin responses are regulated by Elongator at both the transcriptional and translational levels. Notably, the histone acetylation activity of Elongator targets the auxin repressor gene INDOLE-3-ACETIC ACID INDUCIBLE 3 (*IAA3*) and the auxin influx carrier gene LIKE AUXIN RESISTANT 2 (*LAX2*) (Nelissen et al. 2010), whereas its translational activity is required for the synthesis of ARABIDOPSIS PIN-FORMED (PIN) auxin transport proteins (Leitner et al. 2015). Additionally, Elongator regulates transcription and biogenesis of primary microRNAs (miRNAs) (Fang et al. 2015), pointing to yet another mechanism by which it could shape the transcriptome in plants.

Previously, we demonstrated that Elongator regulates skotomorphogenesis, as darkness-grown *elo3–6* seedlings had a shorter hypocotyl compared with the wild type (WT), with cotyledons and apical hooks unaffected (Wołoszyńska et al. 2018). Double-mutant analysis has demonstrated that Elongator is required for hypocotyl elongation under rapid growth conditions, such as darkness or the absence of active phytochrome A or B. A transcriptome analysis of darkness-grown *elo3–6* revealed a pathogen-response-like profile (Rojas et al. 2014) resulting in growth deceleration. Indeed, downregulated genes in *elo3–6* clustered into Gene Ontology (GO) categories forming a growth-controlling network with four main hubs: a circadian clock, regulators of skoto- and photomorphogenesis, diverse hormone response pathways, and primary and secondary cell wall biogenesis. Three downregulated genes—LATE ELONGATED HYPOCOTYL (*LHY*), a homolog of ELONGATED HYPOCOTYL 5 (*HYH*), and LONG HYPOCOTYL IN FAR-RED 1 (*HFR1*)—were identified as Elongator targets for histone acetylation (Wołoszyńska et al. 2018). Additionally, *elo* mutants showed a reduced de-etiolation under red, far-red, or blue light, but no further morphological studies were carried out (Wołoszyńska et al. 2018). Here, we present a detailed analysis of photomorphogenesis defects in *elo3–6* seedlings and their correlation with gene expression alterations.

Photomorphogenesis is driven by massive transcriptome rearrangements resulting in opposite morphological changes in the hypocotyl and the cotyledons. Given the distinct transcriptomes in these two organs, we investigated how the expression of protein-coding and miRNA genes is affected in *elo3–6* mutant hypocotyl and cotyledons during early light-induced seedling development. The rationale for this investigation was the well-supported function of Elongator in the regulation of transcription in plants, including during skotomorphogenesis (Wołoszyńska et al. 2018), and the *elo3–6* phenotype indicating defective photomorphogenesis. Apart from the effects of the *elo3–6* mutation on the transcriptome, we also addressed its consequences on translation in the two organs by analyzing the dependency between mRNA abundance and frequency of codons corresponding to the tRNA anticodons modified by Elongator, hereafter referred to as “Elongator-targeted codons.” We also compared the morphological defects and sensitivity to translation inhibitors of *elo3–6* with another mutant defective in the same tRNA modification, *urm11 urm12*.

## Results

### Elongator complex is required for normal seedling photomorphogenesis

To assess the effects of a nonfunctional Elongator on photomorphogenesis, we compared the phenotypes of *elo3–6* (carrying a homozygous disruption of the *ELO3* gene encoding the catalytic subunit of Elongator) and WT seedlings under constant white light. To confirm that the observed features were indeed due to the Elongator malfunction, we included a complemented line of *elo3–6* [green fluorescent protein (GFP)-ELO3/*elo3–6*] (Nelissen et al. 2010).

**Figure 1:**
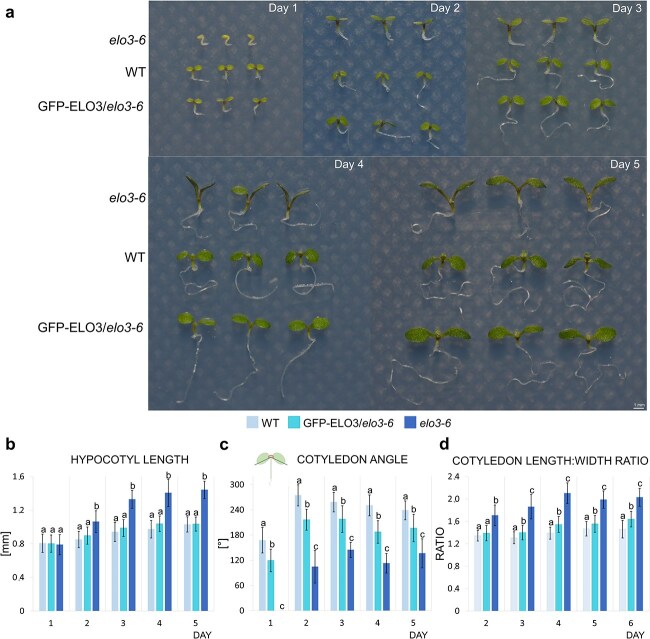
Phenotypic analysis of WT, GFP-ELO3/*elo3–6*, and *elo3–6* seedlings. (a) For each day, three representative seedlings grown *in vitro* under constant white light in vertical position from each line are shown. (b–d) Mean values of indicated parameters. Whiskers indicate standard deviation. The letters above bars indicate groups that are statistically significantly different.

The *elo3–6* seedlings had significantly longer hypocotyls than GFP-ELO3/*elo3–6* and WT seedlings from the second to the fifth day after germination (Fig. 1a and b). The virtually identical hypocotyl lengths in GFP-ELO3/*elo3–6* and WT seedlings prove a full complementation of the *elo3–6* defect.

The cotyledon opening angle in *elo3–6* was significantly lower than in GFP-ELO3/*elo3–6* and the WT (Fig. 1c). This parameter was slightly lower in GFP-ELO3/*elo3–6* than in the WT, indicating that the GFP-ELO3 expression partially compensates for the cotyledon opening defect of *elo3–6*.

The *elo3–6* cotyledons were significantly longer (Supplementary Fig. S1a) and, from the second to the fifth day, narrower than in GFP-ELO3 or the WT (Supplementary Fig. S1b), and consequently had a higher length:width ratio (Fig. 1d).

These results show that Elongator controls hypocotyl growth by suppressing its elongation and is also required for proper cotyledon formation and opening during early photomorphogenesis.

### The Elongator complex regulates distinct gene sets with a greater effect on expression in the hypocotyl than in cotyledons

To assess the role of Elongator in the regulation of photomorphogenesis, we first examined its transcriptional role, given the extensive transcriptome reprogramming upon photomorphogenesis initiation. A comparative RNA-Seq analysis was performed for 4-day-old *elo3–6* and WT seedlings grown under constant white light, separately for the hypocotyl and cotyledons to identify Elongator-regulated genes in each organ.

**Figure 2:**
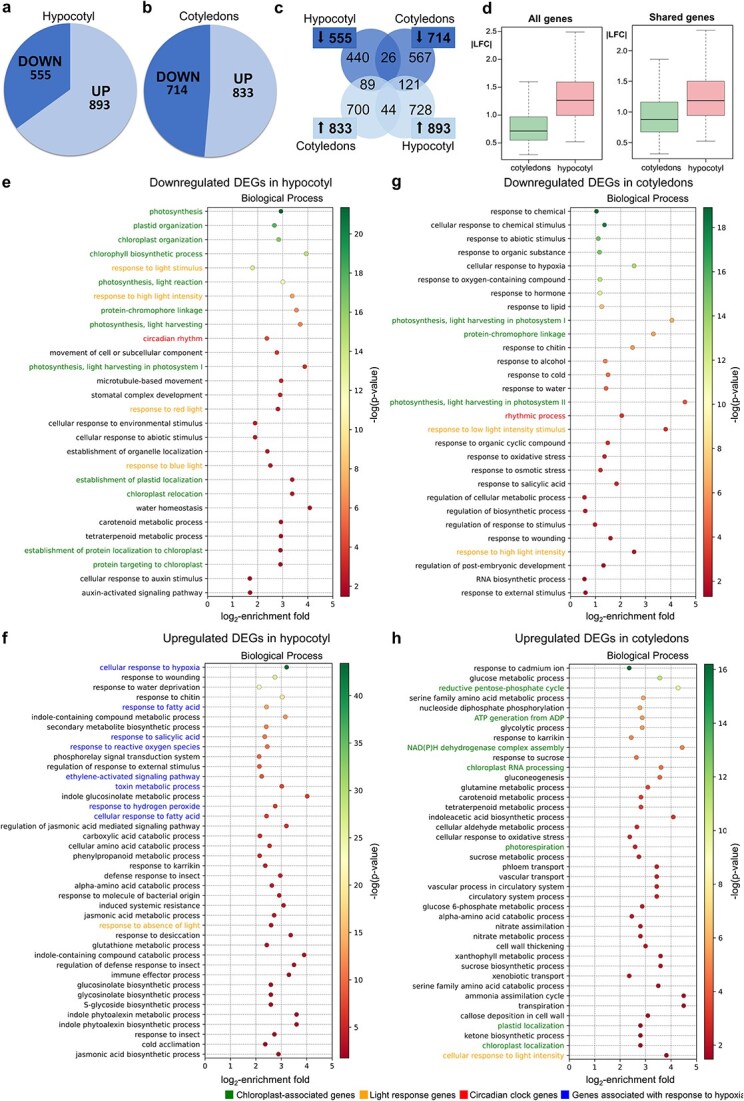
Functional analysis of DEGs identified by comparison of transcriptomes of the *elo3–6* mutant and WT. Down- and upregulation of DEGs found in (a) hypocotyl and (b) cotyledons. (c) Quantitative comparison of hypocotyl and cotyledon DEG sets. (d) Expression difference (|LFC|) of hypocotyl and cotyledon DEGs. The box shows the quartile range, the thick line indicates the median, and whiskers denote the range without outliers. Significantly enriched Biological Process GO categories among (e) downregulated and (f) upregulated DEGs in the hypocotyl, and (g) downregulated and (h) upregulated DEGs in cotyledons. Only nonredundant GO categories are shown. In (e) and (g), only the top 40 categories are shown. Color gradient from green to red indicates decreasing statistical significance, represented by −log(*P*-value).

In hypocotyls, 1448 differentially expressed genes (DEGs) were identified, of which 555 were downregulated and 893 upregulated in *elo3–6* compared with the WT (Fig. 2a). In cotyledons, 1547 DEGs were found, including 714 downregulated and 833 upregulated ones (Fig. 2b). The expression of most of those DEGs changed in only one organ: 440 down- and 728 upregulated DEGs were only found in hypocotyls, and 567 down- and 700 upregulated ones in cotyledons (Fig. 2c).

Given the total number of 2995 DEGs found (1448 in the hypocotyl and 1547 in cotyledons), the overlap of 280 DEGs between the two organs is notably low. Only 70 of those shared DEGs were regulated in the same direction in hypocotyls and cotyledons (26 down- and 44 upregulated) (Fig. 2c). The downregulated ones included the entire NIGHT LIGHT-INDUCIBLE AND CLOCK-REGULATED GENE family (*LNK1–4*), which encodes key factors linking light-dependent gene expression with circadian rhythm; three light-harvesting complex (LHC) genes *LHCB2.1*, *B2.2*, and *B3*; and the auxin repressor gene *IAA3*, a known target of Elongator-dependent histone acetylation (Nelissen et al. 2010) (Supplementary Table S1).

Most of the DEGs shared between the hypocotyl and cotyledons showed opposite expression changes: 121 genes were upregulated in *elo3–6* hypocotyls and downregulated in cotyledons (Fig. 2c), including key circadian clock genes PSEUDO-RESPONSE REGULATOR 5 (*APRR5*), LUX ARRHYTHMO, and GIGANTEA (*GI*) (Supplementary Table S1). Conversely, 89 genes were upregulated in *elo3–6* cotyledons and downregulated in hypocotyls (Fig. 2c); they clustered into the following GO categories: response to light stimulus (BP GO:0009416), photosynthesis (BP GO:0015979), chloroplast organization (BP GO:0009658), and postembryonic development (BP GO:0009791). Notable genes include a negative regulator of seedling photomorphogenesis *BBX25*, GENOMES UNCOUPLED 5 (*GUN5*), and K^+^ efflux antiporters *KEA1* and *KEA3* (Supplementary Table S1).

To assess how strongly the lack of a functional Elongator affects gene expression in the hypocotyl and cotyledons, we calculated the absolute values of their log₂ fold change, i.e. |LFC|, in *elo3–6* mutant versus WT seedlings. We performed several independent comparisons for all combinations of gene sets in the two organs: genes with expression changed only in both organs (shared genes) or genes changed in only one organ, genes regulated oppositely in the two organs or in the same direction, as well as genes with significant or nonsignificant LFC. In all comparisons the median |LFC| values for the hypocotyl were significantly higher than those for cotyledons, by 15%–107% depending on the gene set compared (Fig. 2d and Supplementary Table S2). The results suggest that the absence of a functional Elongator complex has a significantly greater impact on transcription in the hypocotyl than in the cotyledons, despite a similar number of DEGs identified in both organs.

#### In the hypocotyl, Elongator is required for chloroplast functioning, circadian regulation, and response to light and auxin, while its absence initiates response to hypoxia

**Figure 3:**
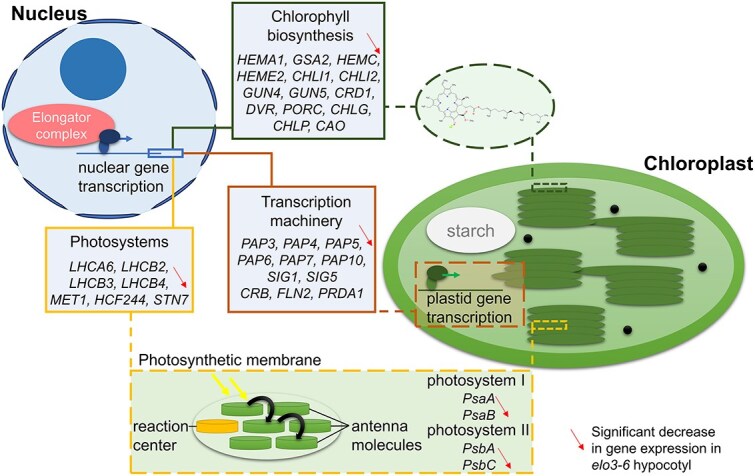
Proposed roles of influence of Elongator transcriptional activity on chloroplast functioning in hypocotyl. Elongator promotes expression of nuclear genes encoding photosystem components and enzymes involved in chlorophyll biosynthesis, as well as genes involved in regulation of transcription in chloroplasts.

The downregulated genes in the *elo3–6* hypocotyl mostly belonged to four groups of enriched GO categories related to chloroplast functioning, response to light, circadian rhythm, and response to auxins (Fig. 2e). Nearly half of them (264) were associated with plastid structure and metabolism and included genes encoding crucial components of the thylakoid membrane: PsaA and B of photosystem I, and PsbA and C of photosystem II or otherwise involved in thylakoid membrane biogenesis (Fig. 3). The two photosystems’ reaction centers are surrounded by LHC proteins, and the expression of both *LHCA* and *LHCB* was decreased in the *elo3–6* hypocotyl, along with several genes encoding regulators of photosystem functioning (Supplementary Table S3). Additionally, genes associated with the formation and activity of the nicotinamide adenine dinucleotide phosphate (NAD(P)H) dehydrogenase complex were also downregulated (Supplementary Table S3). Following this, the downregulated genes were classified to categories related to photosynthesis (BP GO:0015979) and response to light stimuli (BP GO:0009416). Water homeostasis (BP GO:0030104) was the most enriched category, comprising five genes associated with light-harvesting complex proteins (LHCB) and regulators of abscisic acid signaling (Supplementary Table S3).

The downregulated genes from the chlorophyll biosynthetic process (BP GO:0015995) (Fig. 3) category encode a nearly complete set of enzymes of chlorophyll biosynthesis (Supplementary Table S3). Notably among them are the catalytic and regulatory subunits of Mg-chelatase, which also participates in retrograde signaling transmitting the developmental and functional status of plastids to the nucleus (Kindgren et al. 2012).

Plastid genes are transcribed by the bacterial-type plastid-encoded RNA polymerase PEP, which is surrounded by PEP-associated proteins (PAPs) (Pfalz and Pfannschmidt 2013) and guided by sigma-like factors (SIGs) that recognize promoters and initiate transcription (Kanamaru et al. 1999, Chi et al. 2015). The expression of 6 out of 12 *Arabidopsis PAPs*, *SIG1* and *5,* as well as genes encoding other factors necessary for PEP-dependent gene expression was decreased in the *elo3–6* hypocotyl (Fig. 3 and Supplementary Table S3).

Also, the import of proteins to the chloroplast and their movement were likely disturbed in the *elo3–6* hypocotyl, as indicated by the enrichment of the categories of proteins targeting chloroplast (BP GO:0045036) and the establishment of plastid localization (BP GO:0051667) among the downregulated DEGs.

Seventeen downregulated DEGs in the *elo3–6* hypocotyl belonged to the circadian rhythm category (BP GO:0007623), with spectacularly downregulated central oscillator factors *LHY* (10-fold), *CCA1* (nearly 8-fold), and all four *LNK* family members, including *LNK1* and *2*, which are involved in repression of hypocotyl growth (De Leone et al. 2018).

The eighteen downregulated genes from the category cellular response to the auxin stimulus (BP GO:0071365) included the following: two already known targets of Elongator-dependent histone acetylation: *IAA3* and *LAX2* (Nelissen et al. 2010); *PIN3*, encoding the auxin transporter PIN-FORMED 3, the translation of which is known to be dependent on Elongator-mediated tRNA modification (Leitner et al. 2015); as well as Gretchen Hagen 3.6 and 3.17 (*GH3.6* and *3.17*), both involved in auxin homeostasis. The *pin3* mutant fails to elongate its hypocotyl under low-R:FR light due to impaired auxin redistribution (Keuskamp et al. 2010), whereas *GH3.6* overexpression reduces free auxin and shortens the hypocotyl (Nakazawa et al. 2001, Takase et al. 2003), and the *gh3.17* loss-of-function mutant accumulates IAA and shows exaggerated elongation (Zheng et al. 2013). Collectively, downregulation of these genes in the *elo3–6* hypocotyl is likely to disturb auxin homeostasis and thereby promote cell expansion (Supplementary Table S3).

Nineteen downregulated genes in the *elo3–6* hypocotyl clustered into cytoskeleton-related categories, including microtubule (CC GO:0005874) and microtubule-based movement (BP GO:0007018) (Supplementary Table S3). Their decreased expression could lead to microtubule disorganization affecting the deposition of cellulose in the cell wall (Baskin 2005, Lloyd and Chan 2008) and thereby hypocotyl elongation (Kato et al. 2022).

Gene upregulation could constitute a secondary effect of a nonfunctional Elongator. Unlike the downregulated DEGs, the upregulated ones (882 DEGs) formed a dispersed and multithreaded network of Biological Process (BP) GOs (Fig. 2f). However, the Cellular Component (CC) domain indicated a fairly uniform localization of the encoded proteins: 567 DEGs encoded cytoplasmic proteins (CC GO:0005737), and 180 plasma membrane ones (CC GO:0005886).

The most enriched GO categories were the indole-containing compound catabolic process (BP GO:0042436) and indole glucosinolate metabolic process (BP GO:0042343), in agreement with previous studies of the *elo3–6* mutant (Nelissen et al. 2010).

The cellular response to the hypoxia category (BP GO:00714-56) is one with the highest enrichment and statistical significance. Moreover, numerous upregulated genes cluster into GO categories related to changes in metabolism occurring during hypoxia: increased ethylene (ethylene-activated signaling pathway, BP GO:0009873) and the level of fatty acids (BP GO:0070542), accumulation of reactive oxygen species (BP GO:0000302), and activated fermentation leading to accumulation of toxic metabolites (BP GO:0009636) (Xie et al. 2021, Liu et al. 2022) (Supplementary Table S3). Ethylene and lipids (response to lipid, BP GO:0033993) act as signaling molecules, while metabolic adaptation to hypoxia is achieved by hormone action and activation of transcription factors (regulation of transcription, DNA template, BP GO:0006355) and protein kinases. Several upregulated genes encoded proteins involved in hypocotyl elongation and phototropism, and their increased expression agrees well with the hypocotyl elongation observed in *elo3–6* (Supplementary Table S3). Among them was the PIF3 transcription factor, which promotes the expression of *MDP60* to modulate hypocotyl cell elongation in response to light and ethylene signaling (Q. Ma et al. 2018a). PIF3 is also required for submergence-induced and ethylene-dependent underwater hypocotyl elongation (Wang et al. 2019).

To conclude, the GO analysis of the hypocotyl transcriptome demonstrated that the absence of a functional Elongator complex leads to a downregulation of a substantial number of genes associated with chloroplast biogenesis and photosynthesis. The consequent defective functioning of chloroplasts and photosynthesis with impaired oxygen evolution appears to induce a hypoxia-like state evidenced by the upregulation of numerous hypoxia-responsive genes, including transcription factors that activate adaptive mechanisms to counteract this stress.

#### Unlike in the hypocotyl, in cotyledons, Elongator regulates a limited number of genes related to photomorphogenesis

In contrast to the DEGs identified in the hypocotyl, those downregulated in *elo3–6* cotyledons mostly represent enriched GO categories unrelated to photomorphogenesis or development and generally showed low log_2_-enrichment fold, either below or slightly above 1. To focus on the processes most severely affected by the *elo* mutation and expected to be the most relevant to photomorphogenesis, we considered here only the GO categories with log_2_-enrichment fold values above 2 and associated with light harvesting, response to light, or the circadian rhythm (Fig. 2g).

Categories related to photosynthesis or photosystems (BP GO:0019684, GO:0009768, GO:0018298, and GO:0009769, and CC GO:0009522, GO:0009523, and GO:0098807) comprised 17 genes encoding mainly components of LHC proteins of photosystem I and factors contributing to its structure and functioning (Supplementary Table S4). Downregulation of genes encoding LHC proteins suggests that the absence of a functional Elongator complex leads to inefficient harvesting of solar energy by cotyledons. The same is true of a group of photosynthesis-related chloroplast genes, including those encoding subunits of the NADH dehydrogenase-like (NDH) complex and subunits of the ATP synthase complex (Supplementary Table S4). The NDH-dependent cyclic electron transport (CET) is involved in plant response to various environmental stresses, including low light (Ma et al. 2021).

Eighteen downregulated DEGs represent the rhythmic process category (BP GO:0048511), including genes encoding morning and evening loop components and, similarly to the *elo3–6* hypocotyl, the entire LNK1–4 family (Supplementary Table S4).

The response to light stimulus category (BP GO: 0009416) was represented by 44 DEGs, some of which also belong to the photosynthesis and circadian rhythm categories. The most notable genes in this group include *BBX22*, a positive regulator of seedling photomorphogenesis; PHYTOCHROME A (*PHYA*), encoding a crucial factor in light-signal transduction and phototropic responses (Rösler et al. 2007); and *RVE7*, a circadian clock component involved in phytochrome A–mediated cotyledon opening (Kuno 2003). The decreased expression of these genes is in line with the defective photomorphogenic phenotype of *elo3–6* cotyledons (Supplementary Table S4).

In all, 833 DEGs were upregulated in *elo3–6* cotyledons (Supplementary Table S4), of which 400 encode chloroplast components (CCs, GO:0009507), including 108 DEGs of the chloroplast envelope, 125 of stroma, and 84 of thylakoids. Regarding the BP domain, the upregulated DEGs formed a complex multithreaded network comprising processes such as light responses, chloroplast functionality, and energy metabolism (Fig. 2h). The response to light stimulus (BP GO:0009416) category was represented by 69 genes mainly related to photosynthesis, glucose metabolism, and plastids. Among the other genes, components of the SPA (SUPPRESSOR OF PHYTOCHROME A) complex, *SPA3* and *SPA4* (Supplementary Table S4), are particularly interesting in view of their crucial role in the repression of light signal transduction pathways in the dark (Zhu et al. 2008). The photosynthesis (BP GO:0015979) and related categories were also significantly enriched, including NAD(P)H dehydrogenase complex assembly (BP GO:0010275) and ATP generation from ADP (BP GO:0006757). Additionally, the upregulated genes form a network related to carbon (Supplementary Fig. S2) and nitrogen metabolism, including the reductive pentose phosphate (Calvin–Benson) cycle (BP GO:0019253), photorespiration (BP GO:0009853), gluconeogenesis (BP GO:0006094), and the cellular amino acid metabolic process (BP GO:0006520). The Calvin–Benson cycle, photorespiration, and gluconeogenesis are highly interconnected.

Atmospheric carbon dioxide is fixed in the Calvin–Benson cycle using energy produced during the light phase of photosynthesis and stored in ATP and NADPH. Under a low CO_2_-to-O_2_ ratio, photorespiration releases the already fixed CO_2_, protecting plants from photoinhibition damage. The two processes share the RuBisCO enzyme, which acts as a carboxylase in photosynthesis and an oxygenase in photorespiration. In *elo3–6* cotyledons, the expression of several genes encoding key enzymes involved in these processes was significantly increased (Supplementary Table S4).

Products of the Calvin–Benson cycle are used in glycolysis to produce pyruvate, with the concomitant production of ATP and NADH. This provides substrates for mitochondrial respiration and diverse biosynthetic processes, such as gluconeogenesis. Maintaining a balance between glycolysis and gluconeogenesis is crucial for glucose homeostasis, and in *elo3–6* cotyledons genes encoding key enzymes that regulate this metabolic equilibrium were significantly upregulated (Supplementary Fig. S3 and Supplementary Table S4).

The indoleacetic acid biosynthetic process (BP GO:0009684) category was highly enriched in upregulated genes. Given that indoleacetic acid is the primary form of auxin, its increased concentration in the *elo3–6* mutant was expected, potentially correlating with the impaired cotyledon opening angle in *elo3–6*. The upregulation of RIBONUCLEASE J (*RNJ*) (Supplementary Table S4) could further contribute to this phenotype, as RNJ plays a key role in embryogenesis, especially in initiating and maintaining the organization of shoot apical meristems, cotyledons, and hypocotyl (Tzafrir et al. 2004, Chen et al. 2015).

Taken together, the effects of the *elo3–6* mutation on the cotyledon transcriptome, with surprisingly few downregulated photomorphogenesis-related genes, do not explain the strongly affected phenotype of these organs; instead, the functional characteristics of the upregulated genes indicate profound changes in carbon and nitrogen metabolism.

### Genes enriched in Elongator-targeted codons exhibit reduced expression, especially in cotyledons

While the role of Elongator in modifying uridine in tRNA anticodons cannot affect gene expression at the transcription level, the codon–anticodon pairing defects due to a lack of such modification can cause ribosome stalling and degradation of transcripts, especially those rich in the respective codons, thereby indirectly influencing the mRNA level (Powers et al. 2020). To address this possibility, we examined the transcripts expressed in the hypocotyl and cotyledons with respect to the presence of 11 codons recognized by the tRNAs modified by the Elongator. We evaluated the total frequency of these codons (*F*_T_), their relative frequency among synonymous codons to control the specific amino acid usage (*F*_S_), the frequency of at least two (*F*_2_), three (*F*_3_), or four (*F*_4_) such codons occurring consecutively, and the mean distance between them (*D*). The latter four parameters address the distribution of these codons within the coding sequences. It is likely that clusters of these codons rather than separated codons have a stronger effect on disrupting translation and promoting transcript degradation. While genes with increased contribution of codons recognized by tRNAs modified by Elongator have increased *F* (*F*_T_, *F*_S_, *F*_2_, *F*_3_, and *F*_4_) parameters, the *D* value for such genes decreases.

Genes with significantly reduced mRNA levels in the *elo3–6* hypocotyl or cotyledons compared with the WT were significantly enriched in the codons of interest relative to the upregulated genes (Supplementary Table S5). The downregulated genes also contained more clusters of these codons with shorter distances between them. The median values of the studied codon parameters were 3%–209% higher in the downregulated genes relative to the upregulated ones, and the mean distances between these codons were 4%–9% shorter. The differences were statistically significant for all the parameters and for both the cotyledon and the hypocotyl genes (Supplementary Table S5 and Supplementary Fig. S4).

**Figure 4:**
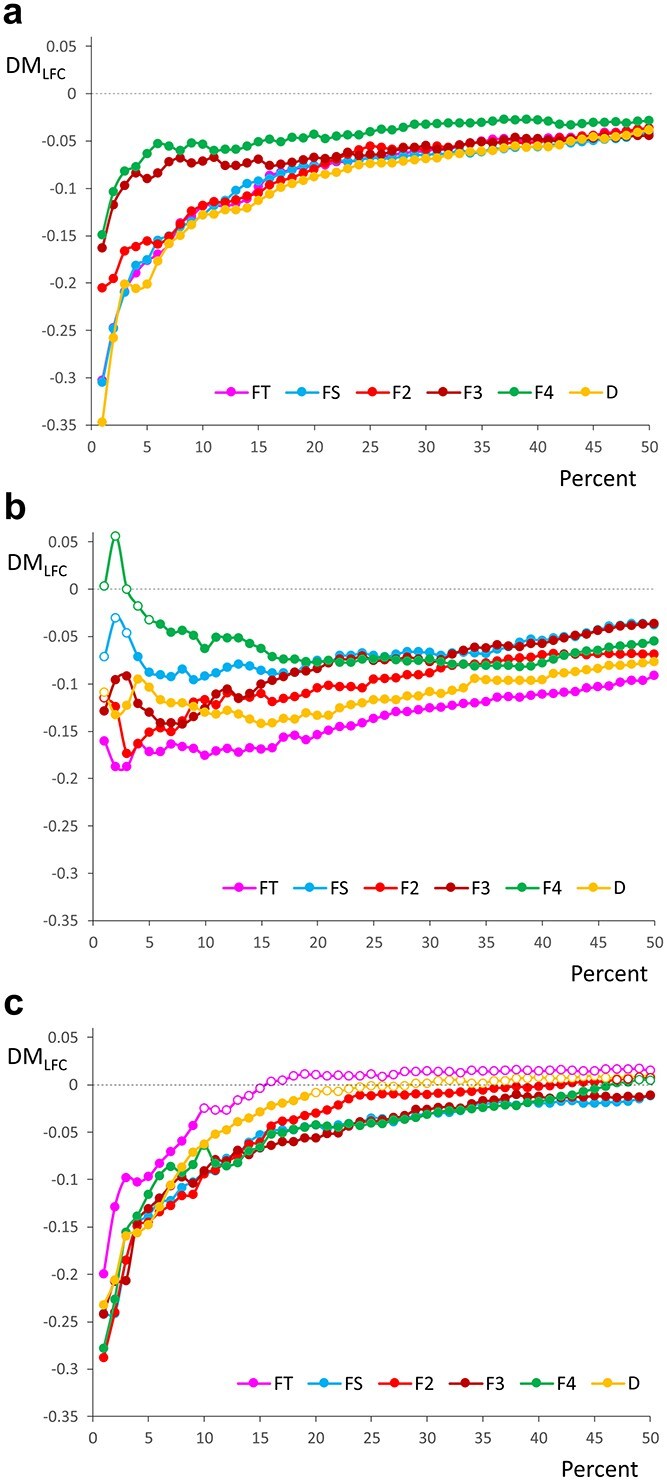
The relationship between the difference in median LFC (DM_LFC_) of genes and various parameters of the Elongator-targeted codons. *F*_T_ is the total frequency of the selected codons among all others, *F*_S_ is the relative frequency of these codons among synonymous ones, *F*_2_, *F*_3_, and *F*_4_ are the fractions of respectively at least two, three, or four codons appearing consecutively, and *D* is the mean distance between the selected codons. Genes were ranked in ascending order according to the value of the codon-related parameters, and then DM_LFC_ was calculated between the bottom and top genes for *F*_T_, *F*_S_, *F*_2_, *F*_3_, and *F*_4_ or between the top and bottom genes for *D* of the ranking. In panels (a) and (b), the genes were considered separately for the cotyledon and hypocotyl, respectively, whereas in (c), the difference was calculated between the LFC of the cotyledon and hypocotyl genes for a given percentage class of the ranking. White circles indicate statistically insignificant differences in LFC between the compared subsets.

We then independently ranked the expressed genes from the two organs separately in ascending order based on the codon-related parameters and selected subsets of 1%, 2%, 3%, and so on, up to 50% of genes from the top (the lowest parameter values) and bottom (the highest parameter values) of this ranking. For each such subset (percentage class), we calculated the median expression changes (LFC). Then, we compared the difference in the median LFCs between the gene subsets selected from the extreme ends of this ranking, progressively moving from the ranking extremes toward the center. Specifically, we compared the subsets with the lowest (LF) versus the highest (HF) codon frequencies and the shortest (SD) versus longest (LD) distances between the codons. The differences in the median LFCs (DM_LFC_) between these gene subsets, i.e. Me(LFC_HF_) − Me(LFC_LF_) and Me(LFC_SD_) − Me(LFC_LD_), are visualized in Fig. 4a and b. The comparisons showed that genes with a higher fraction of the considered codons and shorter in-between distances had lower LFC values than genes with smaller fractions of those codons and wider spacing. The differences in expression level became more pronounced as we compared genes with more extreme parameter values, particularly for cotyledons. The differences were statistically significant for all the comparisons for the cotyledon genes and for most of the comparisons for hypocotyl genes. Some of the comparisons involving extreme parameter values for hypocotyl genes were nonsignificant. Box plots and statistics for gene sets with the most extreme values of the codon parameters are presented in Supplementary Fig. S5 and Supplementary Table S6.

To compare the effect of Elongator-targeted codon frequencies and distributions on the LFC values between hypocotyls and cotyledons, we performed a similar analysis but for the combined sets of DEGS from these two organs. The differences in median LFC values between the corresponding cotyledon and hypocotyl gene subsets decreased with increasing frequency of Elongator-targeted codons and shortening intercodon distances, indicating that gene expression changes (LFCs) in cotyledons were consistently smaller than in hypocotyls (Fig. 4c). The differences in LFCs were statistically significant across all the codon parameters for genes at the ranking extremes. The box plots and statistics for the genes with the most extreme values of the codon parameters are presented in Supplementary Fig. S6 and Supplementary Table S7. Most notably, for the genes highly enriched in the Elongator-targeted codons, the mean and median LFC values were negative for cotyledons and positive for hypocotyls, indicating the opposite effects of the *elo3–6* mutation on the gene expression in the two organs: downregulation in cotyledons and upregulation in hypocotyls.

For a functional characterization of the downregulated genes with the highest frequency and packing of Elongator-targeted codons, we conducted a GO enrichment analysis. Only genes meeting the following criteria were analyzed: a statistically significant LFC ≤ −1 and at least one of the parameters *F*_T_, *F*_S_, *F*_2_, *F*_3_, or *F*_4_ above the 95th percentile (corresponding to a high content of selected codons), or the parameter *D* below the 5th percentile (corresponding to short distances between the analyzed codons) (Supplementary Table S8). These genes turned out to be associated with the auxin pathway and transport, organ growth regulation, response to abiotic stimuli, and the circadian rhythm, in agreement with the previous findings on the GO classification of all the DEGs and the observed *elo3–6* phenotype because all these processes are likely to affect cotyledon growth and opening angle and hypocotyl elongation.

### Elongator complex regulates miRNA expression primarily in hypocotyl

The Elongator complex is known to regulate not only the expression of protein-coding genes but also the transcription and processing of primary miRNA in *Arabidopsis* (Fang et al. 2015). To determine this aspect of Elongator functioning during photomorphogenesis, we compared the expression of genes encoding miRNAs in the *elo3–6* hypocotyl and cotyledons in 4-day-old seedlings grown under constant white light relative to the WT using miRNA sequencing. In the hypocotyl, the abundance of 24 miRNA species was significantly upregulated by the *elo3–6* mutation, and surprisingly no miRNAs were downregulated (Supplementary Fig. S7a). In cotyledons, only four miRNA species were significantly more abundant, and a single miRNA species was significantly downregulated (Supplementary Fig. S7b).

The 24 upregulated miRNA species in the *elo3–6* hypocotyl (Supplementary Table S9) comprised several miRNAs associated with light responses and early development, including miR160, 166, 395, 396, 408, and 858 (Supplementary Table S10). The upregulation of all three members of the miR160 family—miR160a-5p, miR160b, and miR160c-5p—is in line with the hypocotyl elongation observed in the mutant, as miR160 targets and leads to the cleavage of *ARF10*, *16*, and *17* mRNA (Dai et al. 2021). Auxin response factors (ARFs) are transcription factors that inhibit hypocotyl elongation under light conditions (Li et al. 2016). Overexpression of miR160b (*35S::miR160b*) or loss-of-function mutations in *ARF10*, *ARF16*, and *ARF17* result in markedly elongated hypocotyls under light, whereas miR160-resistant *ARF10* lines (carrying synonymous mutations in the miR160 recognition site that prevent *ARF10* mRNA cleavage) develop significantly shorter hypocotyls. These effects persist under brassinosteroid- or gibberellin-deficient conditions and elevated temperature, supporting the role of miR160-mediated *ARF* repression in promoting hypocotyl elongation (Dai et al. 2021). Notably, the level of *ARF10* mRNA was significantly decreased in the *elo3–6* hypocotyl, as determined by RNA-Seq and confirmed by reverse transcription-quantitative polymerase chain reaction (RT-qPCR) (Supplementary Table S3 and Supplementary Fig. S8).

Brassinosteroids upregulate miR395a, which has been shown to suppress *GUN5* expression and thereby regulate chlorophyll synthesis (Lin et al. 2013). The significant downregulation of *GUN5* in the *elo3–6* hypocotyl (Supplementary Table S3) is consistent with the observed upregulation of miR395a, -d, and -e.

In *elo3–6* cotyledons, miR169g-3p was significantly downregulated, and miR319c, miR398c-3, miR472-5p, and miR781a were significantly upregulated (Supplementary Table S9). miR319c targets mRNAs encoding TCP transcription factors (JAW-TCPs) (Supplementary Table S10) which regulate leaf morphogenesis by balancing cell proliferation and differentiation (Palatnik et al. 2003, Nag et al. 2009, Shankar et al. 2023). However, this mode of miR319c involvement in hampering the development of *elo3–6* cotyledons seems unlikely in light of the unaltered levels of TCP-encoding mRNAs found in *elo3–6* cotyledons.

In conclusion, the lack of a functional Elongator complex affects miRNA synthesis much more profoundly in the hypocotyl than in cotyledons. Moreover, only in the hypocotyl did we observe a correlation between an increased abundance of a specific miRNA species and a decreased abundance of its target mRNA encoding a protein relevant to the observed *elo3–6* phenotype.

### Elongator affects thylakoid development

In light of the complex effect of the *elo3–6* mutation on the expression of numerous genes related to photosynthesis, chloroplast biogenesis, etc., as highlighted above, we checked whether and how *elo3–6* affected the chloroplast ultrastructure by performing transmission electron microscopy on ultrathin sections of 6-day-old WT and *elo3–6* cotyledons and hypocotyls. In both organs chloroplasts contained structures characteristic of mature organelles, including thylakoids, starch grains, and plastoglobules. In *elo3–6* cotyledons, the thylakoid system appeared less dense and less organized compared with the WT (Supplementary Fig. S9a, D1). Plastoglobules are present in chloroplasts of both *elo3–6* and WT cotyledons, suggesting no significant differences in their content. In *elo3–6* hypocotyls, chloroplasts seem to contain a reduced number of stacked granal thylakoids (Supplementary Fig. S9b, D2) and a generally less dense thylakoid system (Fig. S9b, D3). More and larger plastoglobules were present in hypocotyl chloroplasts than in the cotyledon ones, but there appeared to be no difference in the plastoglobule abundance between *elo3–6* and WT hypocotyls. Overall, the chloroplast structure appears to be slightly more affected by the *elo3–6* mutation in the hypocotyl than in cotyledons.

### Translational activity of Elongator might contribute to photomorphogenesis in cotyledons

In cotyledons, the expression of relatively few photomorphoge-nesis-related mRNAs and miRNAs was affected by the *elo3–6* mutation, suggesting an involvement of a nontranscriptional mechanism in causing the observed pronounced morphological defects. Considering the known Elongator activities, this additional mechanism is likely to involve modulation of translation. Since the translational activity of Elongator relies on the mcm modification of the tRNA wobble uridine, we investigated the impact of the absence of this modification on photomorphogenesis. To distinguish between the effects of disrupted transcription and translation in *elo3–6*, we analyzed the phenotype of the *urm11 urm12* defective in the activity of enzymes catalyzing thiolation of the Elongator-modified uridine. We assumed that the common phenotypic features of *elo3–6* and *urm11 urm12* likely result from impaired translation due to defective wobble uridine modification in these mutants. RNA-Seq data confirmed that *URM11* and *URM12* expression is unaltered in *elo3–6* cotyledons and hypocotyls, indicating that their misexpression does not explain the *elo3–6* phenotype.

**Figure 5:**
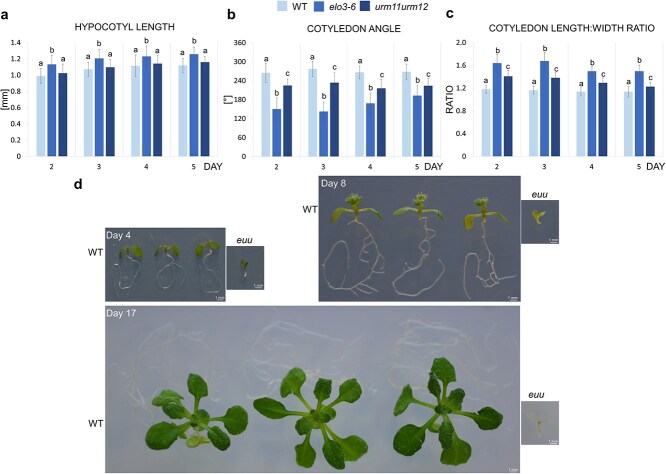
Morphology of the WT, *elo3–6*, *urm11 urm12*, and the *elo3–6 urm11 urm12* (*euu*) triple mutant. Seedlings were grown under constant white light and at times indicated (a) hypocotyl length, (b) cotyledon opening angle, and (c) cotyledon length: width ratios were determined. Mean values with standard deviation are shown. The letters above bars indicate groups that are statistically significantly different. (d) For each day, three representative WT and identified *euu* seedlings grown *in vitro* under constant white light in vertical position are shown.

No significant differences were observed in hypocotyl length between *urm11 urm12* and the WT, in contrast to the significantly longer hypocotyl in *elo3–6* (Fig. 5a), which indicates that an impaired mcm^5^s^2^U_34_ tRNA modification does not lead to hypocotyl elongation during photomorphogenesis and thus supports the exclusively transcriptional role of Elongator in determining hypocotyl growth.

The cotyledon opening angle was significantly lower in *urm11 urm12* compared with the WT, albeit not as low as that in *elo3–6* (Fig. 5b). Also, the cotyledon length:width ratio in *urm11 urm12* was only slightly, but statistically significantly, increased compared with the WT, yet significantly lower than in *elo3–6* (Fig. 5c and Supplementary Fig. S10a and b). These results indicate that an impaired mcm^5^s^2^U_34_ tRNA modification disrupts cotyledon development.

The *euu* triple mutant was generated by crossing *elo3–6* with the *urm11 urm12* double mutant. In the *F*_3_ generation, we identified only three *euu* seedlings, homozygous for all three mutated alleles, at each of the three time points 4, 8, and 17 days after germination. Interestingly, *euu* seedling growth was severely delayed, likely due to synergistic interactions that hinder development (Pérez-Pérez et al. 2009). Although *euu* seedlings were able to germinate, their severe morphological aberrations ultimately resulted in premature death—the 17-day old *euu* seedling was completely transparent. Consequently, a detailed phenotypic characterization of the *euu* mutant was not feasible due to the very limited number of seedlings (Fig. 5d). Notably, all three identified *euu* seedlings had incompletely opened cotyledons, further supporting the importance of Elongator’s translational activity in cotyledon development.

Upon comparing the light-grown *elo3–6* and *urm11 urm12* seedling phenotypes, one must conclude that both the transcriptional and translational functions of Elongator are involved in photomorphogenesis regulation. The relative contribution of these two activities differs markedly between the hypocotyl and cotyledons, the translational activity being particularly important in the latter case.

### Translational Elongator activity is required for cotyledon opening

**Figure 6:**
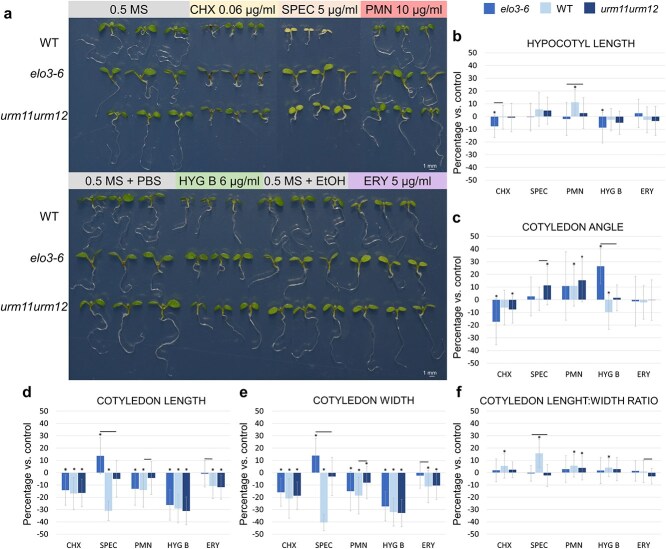
Effects of translation inhibitors on growth of WT, *elo3–6*, and *urm11 urm12* seedlings. Seedlings were grown under constant white light for 5 days in the absence (control) or presence of the indicated translation inhibitors. (a) Three representative seedlings from each line are displayed. (b–f) Bar charts show the mean percentage difference (with standard deviation) to the appropriate control in the phenotypic analyses. The percentage difference for each phenotypic trait was calculated using the formula: $D=100\%\bullet \left({F}_a-\overline{F_c}\right)/\overline{F_c}$, where ${F}_a$ is a value of a given trait under the inhibitor treatment and $\overline{F_c}$ represents the mean value of the given trait in control seedlings. Asterisks indicate statistically significant difference between seedlings grown on the translation inhibitor and control. Horizontal lines indicate statistically significant difference between the mutant and WT.

The similar cotyledon development defects caused by the *elo3–6* and *urm11 urm12* mutations suggest that the translational activity of Elongator has an impact on proper cotyledon growth. If indeed Elongator and URMs affect translation, the responses of *elo3–6* and *urm11 urm12* to translation inhibitors should be similar but that of the WT should be different. To verify this assumption, we assessed the sensitivity of *elo3–6* and *urm11 urm12* seedlings to cycloheximide (CHX), spectinomycin (SPEC), puromycin (PMN), hygromycin B (HYG B), and erythromycin (ERY) (Fig. 6 and Supplementary Fig. S11). These antibiotics bind to various sites on the ribosome, inhibiting different stages of protein synthesis. While CHX is a selective inhibitor of eukaryotic protein synthesis, PMN and HYG B act on both prokaryotic and eukaryotic ribosomes, while SPEC and ERY are inhibitors of prokaryotic protein synthesis only (Mulo et al. 2003). Moreover, CHX, PMN, and ERY interact with the large ribosomal subunit, whereas SPEC and HYG B bind to the small subunit (Fyfe et al. 2012, Wilson 2014, Pellegrino et al. 2021). We used previously defined doses of those inhibitors and compared their effects on the growth of the hypocotyl and cotyledons in *elo3–6*, *urm11 urm12*, and WT seedlings. Both *elo3–6* and *urm11 urm12* seedlings, but especially *elo3–6*, were more tolerant to all the inhibitors than the WT (Fig. 6a). WT seedlings showed significantly delayed growth and even had yellow cotyledons (SPEC treatment), while the mutant seedlings showed little effects.

To gain an insight into the action of Elongator in protein synthesis, we determined whether the effects of translation inhibitors differ between the tRNA modification mutants and the WT. Overall, a significant response to translational inhibitors was mainly observed in *elo3–6* and u*rm11 urm12* cotyledons; however, hypocotyls also showed some differences in their response compared with the WT (Supplementary Fig. S11). We conducted statistical tests to indicate the significant changes between the *elo3–6* and *urm11 urm12* mutants and the WT (Fig. 6b–f, significant changes are marked with horizontal lines). Mutants' hypocotyls were significanlty shorter on CHX and PMN, but the differences did not exceed 14%, and in the case of PMN this difference resulted from the increased length of the WT’s hypocotyl (11%). Notably, HYG B induced a significant increase in the cotyledon opening angle, ≤36% in *elo3–6* and 11% in *urm11 urm12* compared with the WT. Similarly, SPEC increased this angle by 10% in *urm11 urm12* relative to the WT. The largest percentage differences in cotyledon length and width were observed under SPEC treatment: 45% and 26% in length and 54% and 37% in width in *elo3–6* and *urm11 urm12*, respectively. These changes resulted from a dramatic (30%–40%) reduction in length and width in the WT under SPEC and a corresponding increase in *elo3–6*. PMN and ERY also significantly increased the cotyledon length and width (≈10%) in *urm11 urm12* and *elo3–6*, respectively, compared with the WT. The cotyledon length:width ratio decreased most notably in the mutants, compared with the WT, under SPEC (16%–18%) and, to a lesser extent, in *urm11 urm12* under ERY (4%).

Altogether, the above data indicate that cotyledons, rather than hypocotyls, are responsive to translation inhibitors. The most noticeable change not resulting from the response of the WT to translational inhibitors is the substantial increase in the cotyledon opening angle in *elo3–6* grown on HYG B. A similar but less pronounced effect was observed in *urm11 urm12*. This consistent response of tRNA modification mutants to HYG B suggests that both Elongator-dependent tRNA modification and HYG B modulate translation via a shared mechanism involved in the regulation of cotyledon opening.

## Discussion

To determine the contribution of the Elongator complex to photomorphogenesis in *Arabidopsis*, we characterized light-grown *elo3–6* seedlings lacking a functional Elongator and correlated their developmental defects with the effects of the *elo3–6* mutation on gene expression. In the hypocotyl, the *elo3–6* mutation affected the level of numerous mRNA species and miRNAs related to chloroplast functioning, light responses, and the circadian clock, likely leading to exaggerated hypocotyl elongation. In contrast, the transcriptome changes in cotyledons could not explain why *elo3–6* cotyledons were abnormally elongated and narrower and why their opening angle was smaller than in the WT. The seedling responses to translation inhibitors and a bioinformatic analysis of the codon usage in the mRNA species downregulated in *elo3–6* showed that Elongator regulates the development of the two organs during photomorphogenesis, by distinct mechanisms, with its transcriptional role dominant in the hypocotyls and the translation one in cotyledons.

The important role of Elongator in the transcription regulation during hypocotyl elongation in light is supported by the GO categories enriched among genes downregulated or upregulated in the *elo3–6* hypocotyl. The most strongly downregulated genes in *elo3–6* included core circadian clock components *LHY* and *CCA1*, as well as *LNKs*, which link light perception to circadian rhythms and photomorphogenesis. While *lnk1*, *lnk2*, *lnk1 lnk2*, and *lnkQ* quadruple mutants develop long hypocotyls under continuous white light (De Leone et al. 2018), *lhy* and *cca1* exhibit short hypocotyls (Niwa et al. 2007). At first glance, it is puzzling how reduced expression of genes with contrasting individual phenotypes could together contribute to the elongated hypocotyl in *elo3–6*. This may be explained by decreased *LHY*/*CCA1* expression in *lnk1* and *2* and *lnkQ* mutants (De Leone et al. 2018), suggesting that reduced LNK abundance leads to lower *LHY*/*CCA1* transcript levels, and that the hypocotyl length reflects the combined effect of inefficient *LHY*, *CCA1*, and *LNK* expression. Since LNKs interact with RNA polymerase II and the facilitates chromatin transcription (FACT) complex to regulate *TOC1* and *PRR5* (Y. Ma et al. 2018b)—which in turn regulate *LHY*/*CCA1* transcription—downregulation of *LNK* genes in *elo3–6* may alter other transcripts via direct or cascade-like mechanisms, thereby shaping the mutant’s transcriptomic profile. The upregulation of *PIF3* in the *elo3–6* hypocotyl seems associated with its elongated phenotype. In darkness, both *elo* and *pif* mutants develop shorter hypocotyls than the WT, indicating that Elongator and PIFs act in related pathways to promote hypocotyl elongation during skotomorphogenesis (Wołoszyńska et al. 2018). Under light, PIFs are targeted for proteasome-mediated degradation, relieving the repression of photomorphogenesis (Q. Ma et al. 2018). Under light, *pif* mutants maintain shorter hypocotyls than the WT, whereas *elo3–6* exhibits a longer hypocotyl, suggesting that Elongator represses hypocotyl growth independently of PIFs under light.

Nuclear-encoded proteins are imported to the developing chloroplasts to enable thylakoid formation and assembly of photosynthetic complexes (Pribil et al. 2014). The nucleus controls plastid gene expression by so-called anterograde signaling, while plastids emit retrograde signals of their developmental status to regulate the expression of the photosynthesis-associated nuclear genes (*PhANGs*) (Koussevitzky et al. 2007, Woodson et al. 2011, Woodson et al. 2013, Vogel et al. 2014, Martín et al. 2016). The identity of the plastid-related genes downregulated in the *elo3–6* hypocotyl, the decreased chlorophyll levels in *elo* mutants (Falcone et al. 2007, Jun et al. 2022), and, finally, the structural defects of thylakoids all indicate negative retrograde signaling downregulating *PhANG* expression and attenuating photomorphogenesis (Pogson et al. 2015, Chan et al. 2016, Tadini et al. 2020). Loss-of-function mutants in *GUN3* and *HEMERA* (*HMR*/*PTAC12*)—two chloroplast-associated genes that are downregulated in the *elo3–6* hypocotyl—display elongated hypocotyls under light (Mochizuki et al. 2001, Yoo et al. 2019). GUN3 is a canonical component of tetrapyrrole-linked retrograde signaling, whereas PTAC12 is a dual-localized regulator linking phytochrome signaling to chloroplast biogenesis by promoting PEP activity. We hypothesize that the photomorphogenesis defects in the *elo3–6* hypocotyl are caused by retrograde signals provoked by the consequences of the transcriptome changes caused by the absence of an active Elongator. The decreased expression of sigma factors *SIG1* and *SIG5* in the *elo3–6* hypocotyl is in line with the downregulation of genes encoding the photosynthetic reaction centers of PSI and PSII. *SIG1* modulates transcription of *psaA/B and psbA* genes and enables a rapid adaptation of the two photosystems’ activity to light changes (Shimizu et al. 2010). *SIG5* recognizes the promoters of *psbA* and *psbD*, and thereby facilitates the replenishment of PSII proteins damaged in response to stress (Yamburenko et al. 2015). A considerable proportion of genes upregulated in the *elo3–6* hypocotyl represented GO categories hallmarking the response to hypoxia, a state with diminished oxygen levels occurring as a result of submergence when the gas exchange is inhibited. In *Arabidopsis*, hypoxia triggers ethylene signaling, which activates PIF3, stimulating skotomorphogenic growth, and ultimately results in hypocotyl elongation (Wang et al. 2019). In line with this, the upregulation of ethylene-responsive RAP transcription factors in *elo3–6*, assigned to the ethylene-response GO category, is consistent with activation of the ethylene/hypoxia pathway that may be linked to elongation of the light-grown hypocotyl. *RAP2.2* promotes fermentative gene expression and low O_2_ tolerance—its overexpression enhances hypoxia survival, whereas *rap2.2* loss of function reduces survival (Hinz et al. 2010). Similarly, *RAP2.3* overexpression confers hypoxia tolerance (Papdi et al. 2015). Hypoxia can also occur due to inefficient oxygen distribution, high cellular density, or very high metabolism, and can be restricted to individual organs or tissues (Loreti and Perata 2020). Very young seedlings experience oxygen deficit shortly after germination in water-logged soil, which, together with the absence of light, promotes skotomorphogenic growth. Therefore, next to light perception, oxygen sensing is proposed as a key factor necessary for optimal timing of the transition from skoto- to photomorphogenesis (Abbas et al. 2015). Physiological hypoxia usually does not occur in autotrophic tissues since they produce photosynthetic oxygen; however, we propose that the disturbed chloroplast biogenesis in the *elo3–6* hypocotyl leads to transcriptome adjustments typical for hypoxia, and thus to an excessive elongation of the hypocotyl.

Interestingly, the effect of the *elo3–6* mutation on gene expression was almost completely distinct between the hypocotyl and cotyledons. Unlike in the hypocotyl, the transcriptome alterations in *elo3–6* cotyledons were not consistent with their severe morphological defects indicating abnormal photomorphogenesis. Considering that genes related to photosynthesis are activated in cotyledons early during photomorphogenesis (Mansfield and Briarty 1996), we expected their decreased expression in the *elo3–6* mutant. Indeed, some genes of the light phase were downregulated and grouped in a well-enriched GO cluster, while genes related to the dark phase of photosynthesis and carbon metabolism, including photorespiration, were upregulated, pointing to a compensatory mechanism stimulating photorespiration in response to the inactivation of photosynthetic CET (Chen et al. 2023). Similar carbon metabolism–related pathways had highly altered fluxes in the *reil1-1reil2-1* double mutant with defective ribosome biogenesis (Pries et al. 2021). Considering that *elo* mutations, in addition to their effect on transcription, also result in defective modification of certain tRNAs, and thus affect protein biosynthesis, the similarities between the *reil1-1reil2-1* and *elo3–6* mutants seem highly relevant to the understanding of how the absence of an active Elongator could affect metabolism through a mechanism different from transcriptome modulation. Although these changes are not directly linked to the cotyledon phenotype, they highlight broader metabolic alterations in *elo3–6*, consistent with defects in protein synthesis. Additionally, the median |LFC| values for hypocotyls were significantly higher than those for cotyledons across the various gene sets compared, suggesting that the absence of a functional Elongator complex has a significantly greater impact on transcription in the hypocotyl than in cotyledons, despite the similar numbers of DEGs identified in both organs. A comparable pattern of more extensive transcriptomic changes in the hypocotyl has been reported in *Arabidopsis*: shade- and ethylene-induced genes were more numerous in the hypocotyl than in cotyledons (Das et al. 2016, Kohnen et al. 2016); the *hda9-1* mutant (lacking histone deacetylase 9) shows the same pattern under light and shade and exhibits attenuated hypocotyl elongation in shade (Nguyen et al. 2023); and more long noncoding RNAs are expressed in the hypocotyl than in cotyledons (García-López et al. 2024).

As for the mRNA transcriptome, the alterations in miRNA abundance in the *elo3–6* hypocotyl also seem to be relevant to its photomorphogenesis defects, while those in cotyledons are only marginally so. The number of upregulated miRNAs was much higher in the hypocotyl and, for six miRNA species, was connected with a decrease of the abundance of their known target mRNAs. Our data contradict the previously reported positive regulation of miRNA biogenesis by the Elongator complex (Fang et al. 2015); this discrepancy could reflect differences in the design of the two studies, including the plant growth conditions, organs used for RNA isolation, and techniques of miRNA identification.

In contrast to the hypocotyl, in the case of cotyledons we were unable to find a convincing transcription-based explanation for their clearly disturbed photomorphogenesis. Taking into account the contribution of Elongator to the modulation of translation, we evaluated its impact on photomorphogenesis, especially in cotyledons, by comparing the defects of *elo3–6* seedlings with those of the *urm11 urm12* mutant, which lacks the activity of enzymes that, similarly to Elongator, are essential for the wobble uridine modification required for efficient translation. The decreased angle between cotyledons and their elongated shape, common to both mutants, indicated that indeed Elongator regulates photomorphogenesis at the translation stage of gene expression. This conclusion is supported by the phenotype of GFP-ELO3/*elo3–6* seedlings, showing a full restoration of hypocotyl length, but only partial restoration of cotyledon development. This phenotype can be explained by the two Elongator activities differently contributing to the development of the hypocotyl and cotyledons showing varying degrees of restoration by the GFP-ELO3 construct, with better restoration of the transcription activity crucial in the hypocotyl. The more important role of Elongator in the regulation of gene transcription in the hypocotyl than in cotyledons is additionally supported by the significantly stronger impact of the *elo3–6* mutation on the abundance of organ-specific mRNAs in the hypocotyl. To further improve our understanding of the importance of Elongator’s translational activity during photomorphogenesis, we attempted to generate the triple *euu* mutant. Despite the relatively mild effects of *elo3–6* and double *urm11 urm12* mutations, their combination appeared synergistic and resulted in seedling lethality. This severe *euu* phenotype indicates that at least one modification, either mcm^5^ or s^2^, in wobble uridines of tRNAs targeted by Elongator and URMs is required for plant viability. Similar lethality of mutants lacking both mcm^5^ and s^2^ modifications has previously been shown in *Saccharomyces cerevisiae* (Björk et al. 2007) and *Caenorhabditis elegans* (Chen et al. 2009).

Studying the sensitivity of *elo3–6* and *urm11 urm12* to diverse inhibitors of translation allowed us to compare the input of the Elongator-dependent tRNA modification to the development of the hypocotyl and cotyledons. At first glance, the *elo3–6* and *urm11 urm12* seedlings appeared resistant to various translation inhibitors, which is commonly linked to deficient ribosome biogenesis (Abbasi et al. 2010, Rosado et al. 2010, Hang et al. 2014). However, our analyses showed that some translation inhibitors enhanced the effects of the *elo* and *urm* mutations, but most of them counteracted the mutation effects, mimicking phenotypic reversion, with statistical significant differences > 10% when comparing treated to control samples or mutants with the WT (Fig. 6 and Supplementary Fig. S11). The most pronounced effect was caused by HYG B, which partially restored the cotyledon opening angle in *elo3–6* and to a lesser extent in *urm11 urm12*. Thus, this inhibitor effectively mimicked the Elongator function in translation. Indeed, the mcm^5^s^2^U_34_ tRNA modification performed by Elongator and URMs strengthens the tRNA binding at the ribosomal A site, and so does HYG B by binding to the small ribosomal subunit next to the A site (Brodersen et al. 2000). In WT plants with properly modified tRNAs the stabilizing effect of HYG B prevents the translocation of tRNA to the P-(peptidyl) site (González et al. 1978, Brodersen et al. 2000, Borovinskaya et al. 2008), whereas in *elo3–6* and *urm11 urm12* the interplay of the destabilizing effect of a lack of U_34_ modification with the stabilizing action of HYG B more-or-less cancels each of these effects and effectively reverses the consequences of those mutations, as evidenced by the restoration of the cotyledon opening angle.

SPEC also slightly increased the angle in *urm11 urm12*. Moreover, SPEC caused a significant elongation of *elo3–6* cotyledons, aligning with the mutation’s effect, while simultaneously inducing cotyledon shortening in the WT. Interestingly, this inhibitor led to cotyledon widening in *elo3–6*, in contrast to the mutation’s effect. Although SPEC, like HYG B, is an aminoglycoside that binds the small ribosomal subunit, interfering with peptidyl tRNA translocation (Mulo et al. 2003, Wilson 2014), it only affects prokaryotic-type translation—in this case that in organelles, without directly affecting cytoplasmic protein synthesis. Therefore, the effect of SPEC on some phenotypic defects of the *elo3–6* or *urm11 urm12* mutants cannot be easily explained and needs further investigation.

The wobble uridine modification catalyzed by Elongator is essential for accurate codon–anticodon pairing and optimal translation speed and accuracy (Agris 2015, Väre et al. 2017). Thus, its disruption should impair translation, making it both slower and more error-prone for codons recognized by the tRNA species modified by Elongator. Notably, transcripts enriched in those codons were less abundant in *elo3–6*, most likely due to their enhanced degradation via mRNA-surveillance mechanisms, e.g. no-go decay (NGD) (Doma and Parker 2006, Navickas et al. 2020) or processes similar to nonsense-mediated mRNA decay (NMD) (Chang et al. 2007). The decrease in the transcript level of genes rich in these specific codons was more pronounced in cotyledons than in the hypocotyl, in agreement with the stronger translational role of Elongator in the former.

Genes with a significantly lower expression in *elo3–6* and enriched in codons recognized by Elongator-modified tRNAs turned out to be involved in the auxin pathway and transport of auxin, organ growth regulation, response to abiotic stimuli, and the circadian rhythm. Thus, the tRNA modification activity of Elongator could regulate the efficiency of synthesis of proteins responsible for specific processes. Such proteins could also prepare plants to abiotic stresses, such as drought, salinity, and temperature extremes or regulate the physiological rhythm that coordinates the plant’s growth and development with environmental light/dark cycles, including photosynthesis, hormone signaling, and flowering. These results point to a previously unrecognized mode of Elongator-mediated regulation of gene expression. Besides facilitating transcription through its epigenetic activity and modulating the efficiency of translation via tRNA modification, Elongator can also affect transcript stability.

**Figure 7:**
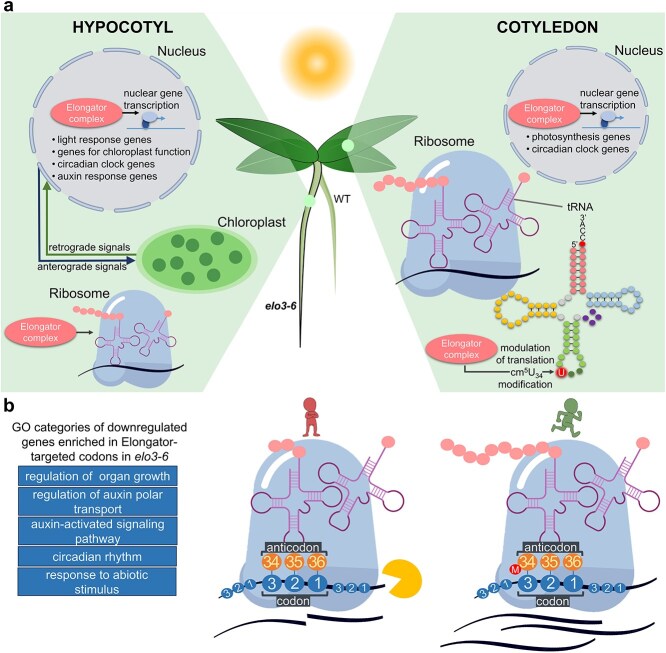
Proposed model of elongator functions in photomorphogenesis. (a) In the hypocotyl, Elongator suppresses elongation mainly through its transcriptional activity in the cell nucleus. Elongator promotes expression of genes associated with light responses, chloroplast functionality, the circadian clock, and auxin responses. Among these genes are those involved in anterograde signaling, which shape chloroplast functioning. In cotyledons, Elongator acts mainly by introducing modification of the wobble uridine in several tRNA species in the cytoplasm, which modulates translation. Its transcriptional role is of lesser importance in cotyledons. (b) The loss of Elongator-dependent wobble uridine modification in tRNA impairs translational efficiency and accuracy, which leads to more frequent degradation and reduced abundance of transcripts enriched in codons recognized by the modified anticodons. GO categories of downregulated genes carrying Elongator-targeted codons are enriched in *elo3–6*, especially in cotyledons. The *elo3–6* seedling is outlined in bold—its cotyledons are hyponastic and less opened, and the hypocotyl is elongated compared with the WT.

Our results document an organ-specific impact of transcrip-tion- and translation-related functions of Elongator in gene expression, as presented in Fig. 7. Although the observed difference in the mode of Elongator’s impact on the hypocotyl versus cotyledons warrants further detailed studies, it should be stressed that the comparison of the effects of the *elo3–6* and *urm11 urm12* mutations on hypocotyl elongation excludes the possibility of an Elongator-mediated translational regulation of this process. Notably, all the earlier studies cited above reporting an essential role of translational regulation during photomorphogenesis were based on analyses of RNA samples isolated from whole seedlings or their entire aerial parts mostly comprised of cotyledons, and therefore they did not address the hypocotyl as such. We cannot yet determine the exact contribution of the various regulatory functions performed by Elongator in the hypocotyl and cotyledons, but we can unequivocally conclude that Elongator differentially controls photomorphogenesis in these two organs of *Arabidopsis* seedlings.

## Materials and Methods

### Plant material

The wild-type and mutant *A. thaliana* plants were of the Columbia-0 ecotype. The mutant lines included the *elo3–6* mutant (GABI-KAT collection code GABI555_H06) (Nelissen et al. 2010); the *urm11 urm12* double mutant, obtained by crossing of the *urm11-1* (SALK_024513.19.30.x) and *urm12-2* (SALK_070672.47.90.x) single mutants; the *elo3–6 urm11 urm12* (*euu*) triple mutant, generated by crossing *elo3–6* with the *urm11 urm12* double mutant (genotyping conditions are described in Methods S1); and the GFP-ELO3/*elo3–6* transgenic line obtained by transforming *elo3–6* with a GFP-ELO3 construct, enabling the expression of ELO3 protein fused to the GFP tag under the 35S promoter (Nelissen et al. 2010).

### Growth conditions and phenotypic analysis

Sterile seeds were sown on basal-solid half-strength Murashige and Skoog (0.5 MS) medium (Murashige and Skoog 1962) without sucrose and stratified at 4°C for 72 h. The seeds were then exposed to constant white light at 45 μmol m^−2^ s^−1^, 85% humidity, and 21°C in vertical position in a PHCbi Versatile Environmental Test Chamber with Panasonic FL40SS ENW/37 bulbs. Seedlings were analyzed as specified below for hypocotyl length, cotyledon length and width, and cotyledon opening angle using ImageJ software. The measurements were taken from at least 20–30 seedlings per genotype for each parameter. For hypocotyl length and cotyledon opening angle, the seedlings were placed on 1% (w/v) agarose and photographed. The cotyledon length and width were measured on dissected cotyledons placed between two chilled slides and photographed.

### Seedling response to translation inhibitors

Seedlings were germinated and grown on basal medium supplemented with one of the following translation inhibitors: CHX (0.06 μg ml^−1^), SPEC (5 μg ml^−1^), PMN (10 μg ml^−1^), HYG B (6 μg ml^−1^), and ERY (5 μg ml^−1^). The inhibitor concentrations used were determined experimentally by testing a range of doses and based on literature data. Controls for HYG B and ERY contained equivalent amounts of phosphate-buffered saline (PBS) and ethanol (EtOH), respectively, added to the medium.

### Transmission electron microscopy

For thin-section electron microscopy, hypocotyls and cotyledons of 6-day-old seedlings were collected and stored in 2.5% glutaraldehyde. Prior to dehydration, samples were rinsed in 0.2 M sodium cacodylate (pH 7.2) and fixed in 2% aqueous osmium tetroxide for 2 h. After that, the samples were washed in distilled water, dehydrated through an ethanol series (30%, 50%, 70%, 90%, 96%, and 99.8%), and embedded and polymerized for 24 h at 60°C in Agar 100 epoxy resin (Agar Scientific). Ultrathin (60–80 nm) sections were cut with a diamond knife on a Leica EM UC7 Ultramicrotome. The sections were stained with 2% uranyl acetate and examined in a JEOL 1200EX transmission electron microscope operated at 80 kV (Spurr 1969, Allen et al. 1988); between 35 and 130 images per tissue were analyzed.

### RNA-Seq

Total RNA was extracted separately in three biological replicates from cotyledons and hypocotyls of 4-day-old seedlings using the RNeasy Plant Mini Kit (QIAGEN) with slight modifications: incubation of samples with the RLT buffer at 56°C for 1 min, two additional washes of the columns with 500 and 300 μL of RPE buffer, and DNase I treatment. The isolated RNA was used for the construction of RNA-Seq libraries by the company Eurofins. The library preparation comprised purification of poly-A-containing mRNA molecules, fragmentation, random-primed cDNA synthesis, adapter ligation, and PCR amplification. Then, a 150-bp paired-end sequencing run was performed on an Illumina platform. The bioinformatic pipelines used for data elaboration are described in Methods S2. Differentially expressed genes between *elo3–6* and the WT were identified using the false discovery rate (FDR)-adjusted *P*-value cutoff of 0.05, an LFC threshold of 0.5, and a minimum mean count of 100. Ontological analysis was conducted using Dicots PLAZA version 5.0. The significance of GO enrichment analysis was determined at a *P*-value cutoff of 0.05 with Bonferroni correction in the Biological Process and Cell Component domains. The filtered hierarchy option was applied to minimize redundancy. The expression of selected genes was verified by RT-qPCR (Supplementary Tables S3, S5, and  S11; Methods S3; and Supplementary Fig. S8).

### miRNA-Seq

RNA fractions enriched in small RNAs were extracted in three biological replicates separately from cotyledons and hypocotyls of 4-day-old seedlings using miRNeasy Mini Plant (QIAGEN) according to the original protocol and used for the construction of miRNA libraries by the company Genomed S.A. Then, 50-bp single-end sequencing was performed on an Illumina platform. The bioinformatic pipeline used for data elaboration is described in Methods S4. Differentially expressed miRNA genes were identified with the FDR-adjusted *P*-value cutoff of 0.05. Only miRNAs that have been experimentally validated in miRBase were investigated in detail. Targets of the differentially expressed miRNAs were predicted with the use of miRanda and extracted from published experimental evidence.

### Codon frequency analyses

Several parameters related to codons recognized by the tRNAs whose wobble uridine in the anticodon is modified by Elongator were calculated for the coding sequences of DEGs. These codons include the following: TTA (Leu), GTA (Val), TCA (Ser), ACA (Thr), GCA (Ala), and CCA (Pro) with ncm^5^U_34_; GGA (Gly) and AGA (Arg) with mcm^5^U_34_; and CAA (Gln), GAA (Glu), and AAA (Lys) with mcm^5^s^2^U_34_. The following parameters were calculated: the total frequency of the Elongator-targeted codons among all the codons (*F*_T_), the relative frequency among synonymous codons (*F*_S_), the fraction of at least two (*F*_2_), three (*F*_3_), or four (*F*_4_) codons appearing consecutively, and the mean distance between such codons given in codons (*D*). The DEGs under investigation were ranked in ascending order of each parameter and defined percentiles from both the top and bottom of the ranking were compared for their LFC values.

### Statistical analyses

The phenotypic measurements of the WT and mutant seedlings were checked for consistency with a normal distribution using the Shapiro–Wilk test. For normally distributed data, the analysis of variance test was performed followed by a *post hoc* NIR test to determine the significance of differences between the groups. Otherwise, the nonparametric Kruskal–Wallis test was applied. In both analyses, *P* < 0.05 was assumed to indicate statistical significance. The tests were performed in Statistica.

The following statistical procedures were applied to determine the significance of differences in gene expression between organs, the correlation of the codon parameters with gene expression, and the seedling response to antibiotics. The Shapiro–Wilk test (up to 5000 cases) or Anderson–Darling test (more than 5000 cases) was used to determine whether the analyzed variables follow a normal distribution. If so, two groups were compared using Welch’s *t*-test. Otherwise, its nonparametric counterpart, the Wilcoxon–Mann–Whitney test was applied. When several hypotheses were tested, the Benjamini–Hochberg correction of *P*-values was applied to control for the FDR. The analyses were performed in R software 4.3.3 (R Core Team 2024).

## Supplementary Material

pcp-2025-e-00159-File009_pcag005

pcp-2025-e-00159-File010_pcag005

pcp-2025-e-00159-File011_pcag005

## Data Availability

Raw RNA-Seq and miRNA-Seq data have been deposited at the Sequencing Read Archive (SRA) database at the National Center for Biotechnology Information (https://www.ncbi.nlm.nih.gov/) under accession numbers: PRJNA1114891 and PRJNA1231046.
