# Supplementary material for: Elongator complex differentially regulates transcription and translation in the hypocotyl and cotyledons during early light-dependent Arabidopsis development
Source: Plant Cell Physiol. 2026 Jan 16;67(5):826–46. doi: 10.1093/pcp/pcag005 (PMC13227157; doi:10.1093/pcp/pcag005)
Supplement: pcp-2025-e-00159-File009_pcag005 [file pcp-2025-e-00159-file009_pcag005.pdf]

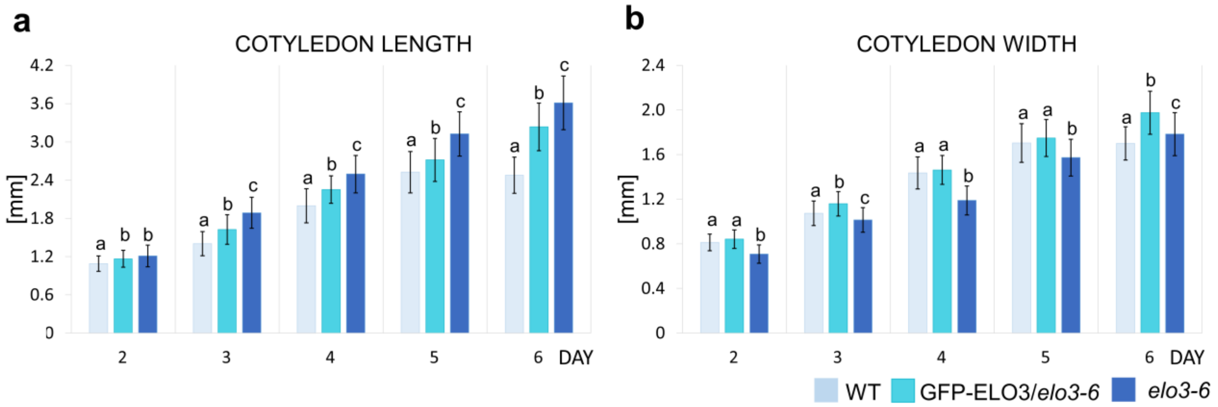

Fig. S1 Cotyledon dimensions in WT, GFP-ELO3/*elo3-6* and *elo3-6* seedlings. Seedlings were grown under constant white light and at times indicated (a) cotyledon length and (b) width were determined. Mean values with standard deviation are shown. The letters above bars indicate groups that occurred statistically significant different.

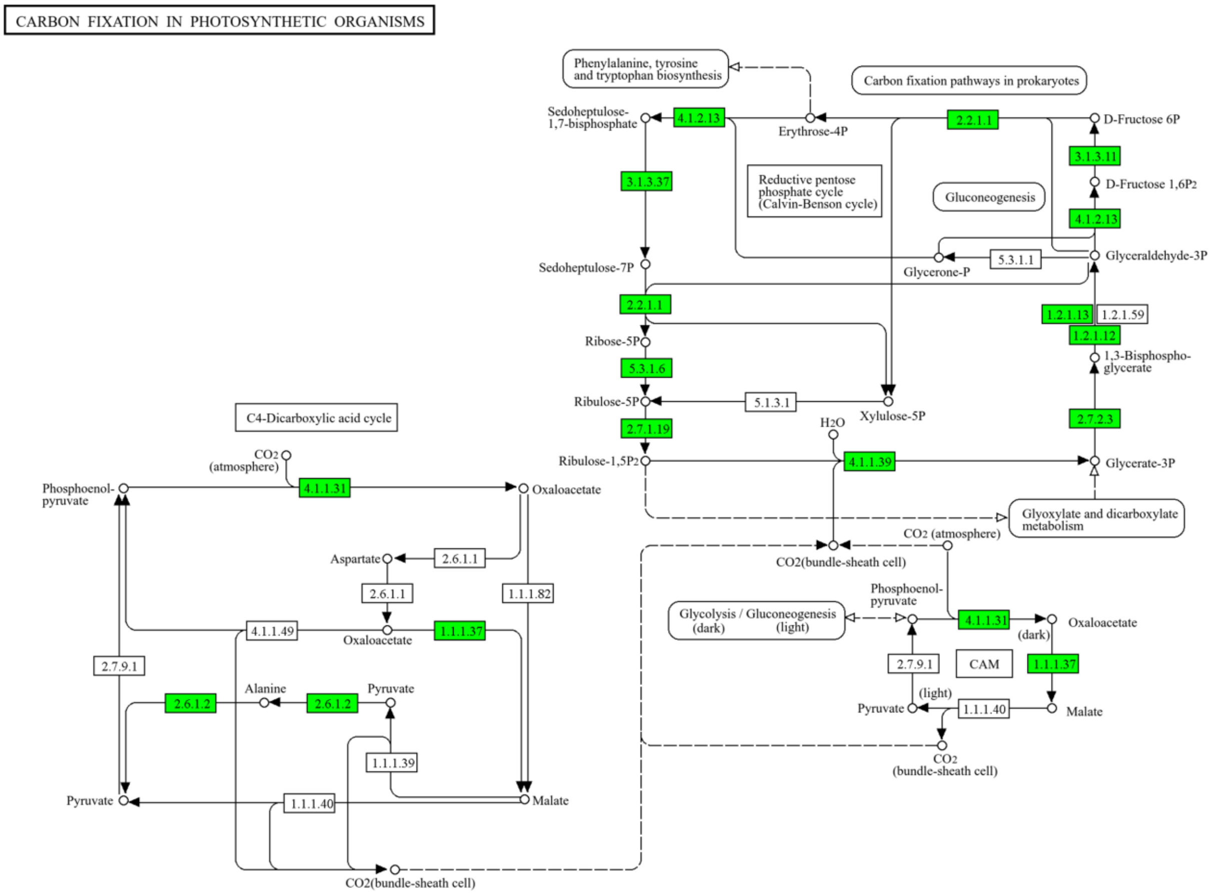

Fig. S2 Carbon fixation pathways in photosynthetic organisms. Proteins corresponding to upregulated genes in *elo3-6* cotyledons are highlighted in green. Source KEGG Mapper – Color.

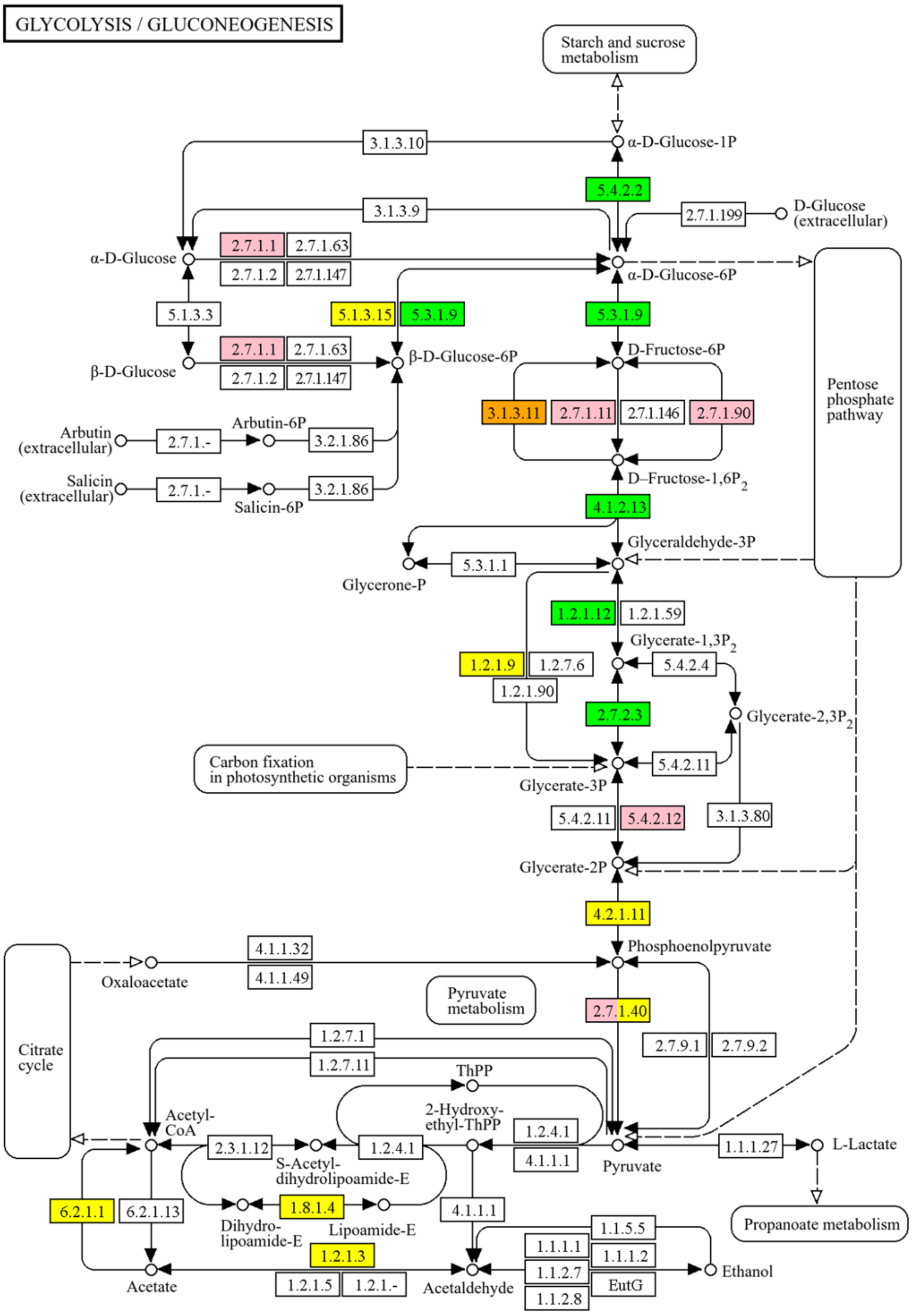

Fig. S3 Glycolysis and gluconeogenesis pathways. Enzymes corresponding to upregulated genes in *elo3-6* cotyledons are highlighted in different colors: pink represents glycolysis-specific genes, orange represents gluconeogenesis-specific genes, green represents genes shared by both pathways, and yellow represents genes indirectly involved in these processes. Source KEGG Mapper – Color.

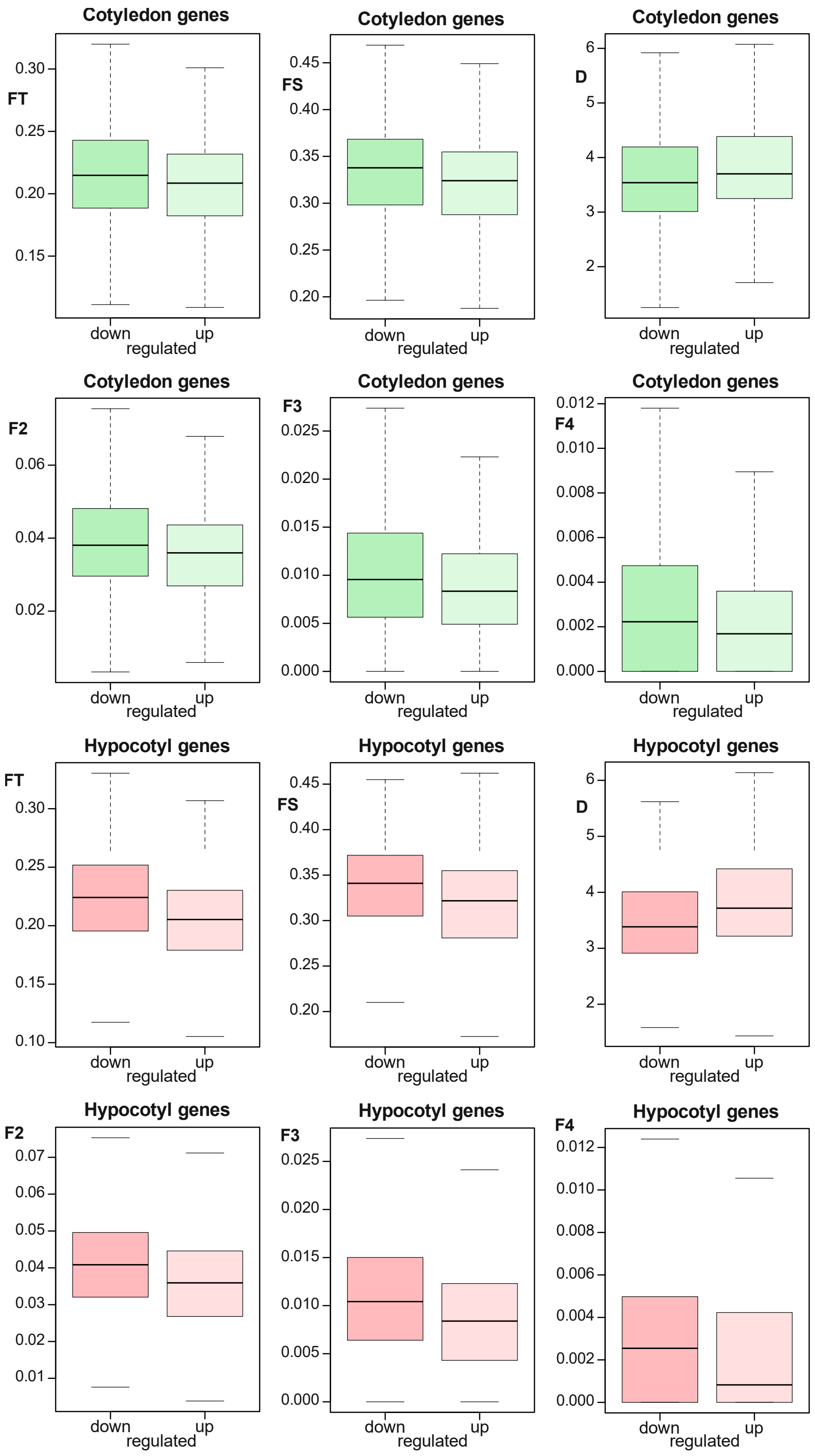

Fig. S4 Parameters related to Elongator-targeted codons in differentially expressed genes (DEGs) between *elo3-6* and WT in hypocotyl and cotyledons. FT is the total frequency of the selected codons among all others, FS is the relative frequency of these codons among synonymous ones, F2, F3 and F4 are the fractions of respectively at least two, three or four codons appearing consecutively, D is the mean distance between the codons. Thick line indicates the median, box shows quartile range, and whiskers denote range without outliers.

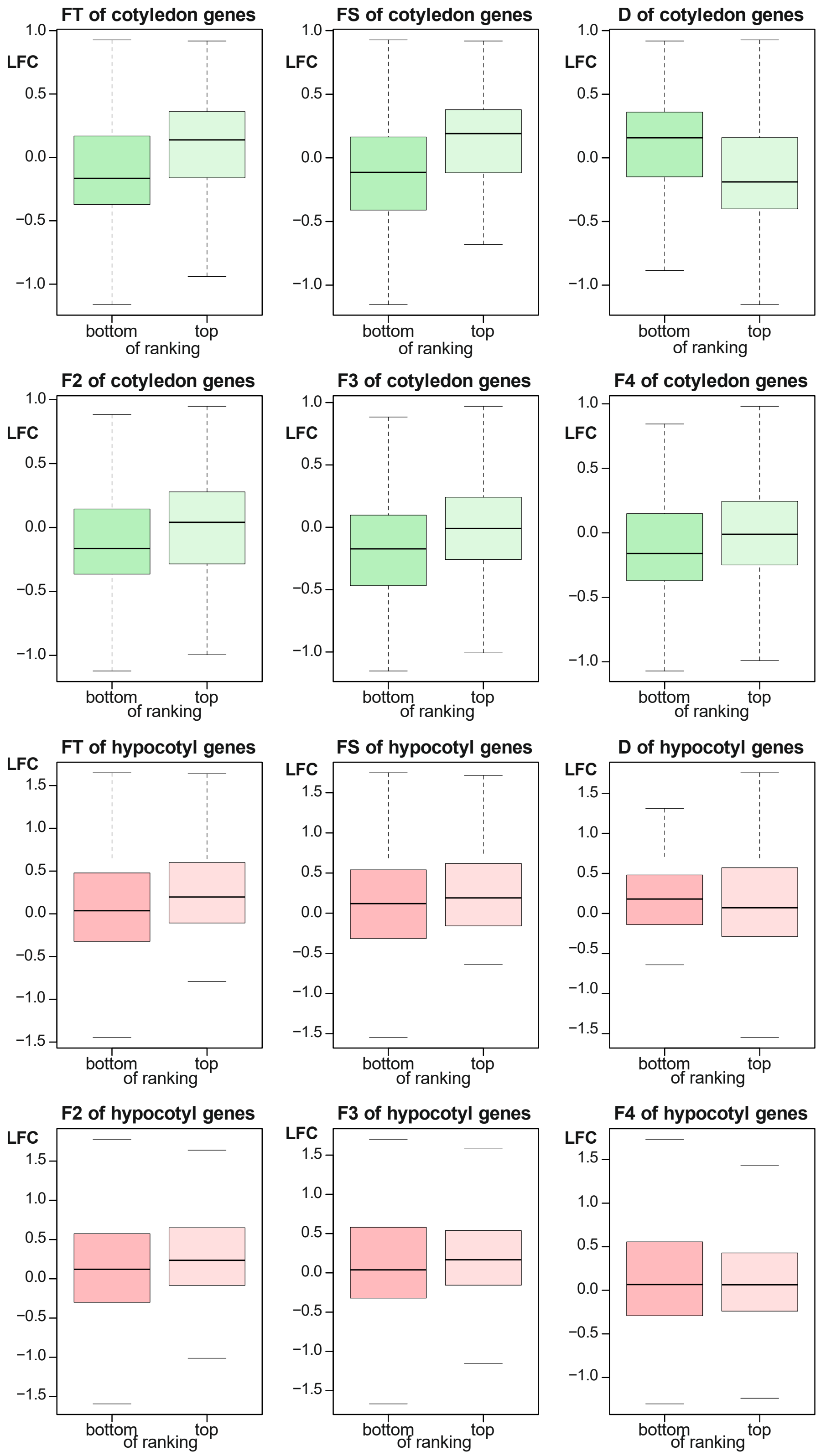

Fig. S5 Expression level difference between *elo3-6* and WT ($\log_2$ fold change, LFC) for genes with the extreme values of the Elongator targeted-codons parameters, where DEGs identified in hypocotyl and those from cotyledons were analyzed separately. Top one percentile genes were compared with bottom one percentile genes from ascending rankings according to the codon parameters. FT is the total frequency of the selected codons among all others, FS is the relative frequency of these codons among synonymous ones, F2, F3 and F4 are the fractions

of respectively at least two, three or four codons appearing consecutively, D is the mean distance between the codons. Thick line indicates the median, box shows quartile range, and whiskers denote range without outliers.

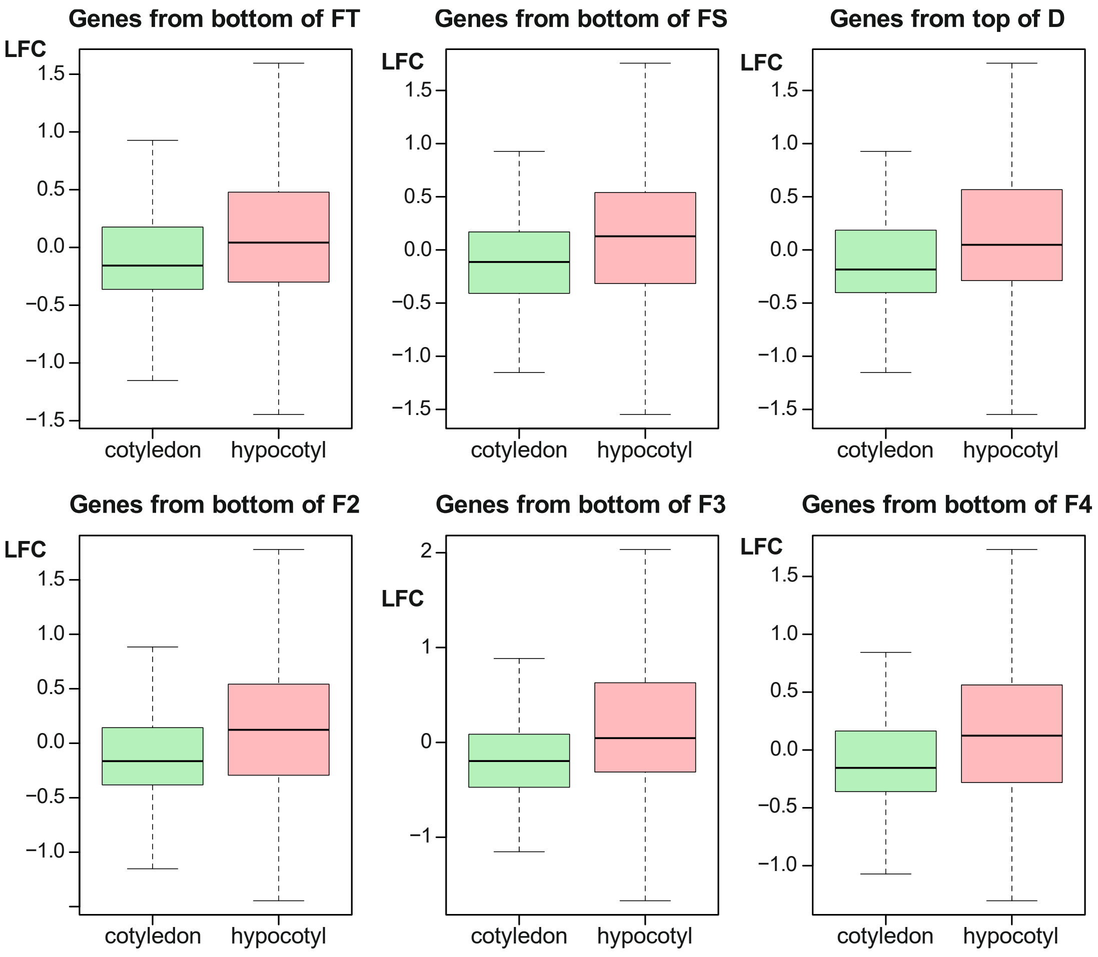

Fig. S6 Expression level difference between *elo3-6* and WT ($\log_2$ fold change, LFC) for genes with the extreme values of the Elongator targeted-codons parameters, where DEGs identified in hypocotyl and those from cotyledons were analyzed as one set. Cotyledon genes were compared with hypocotyl genes for top one percentile genes and bottom one percentile genes from ascending rankings according to the codon parameters. FT is the total frequency of the selected codons among all others, FS is the relative frequency of these codons among synonymous ones, F2, F3 and F4 are the fractions of respectively at least two, three or four codons appearing consecutively, D is the mean distance between the codons. Thick line indicates the median, box shows quartile range, and whiskers denote range without outliers.

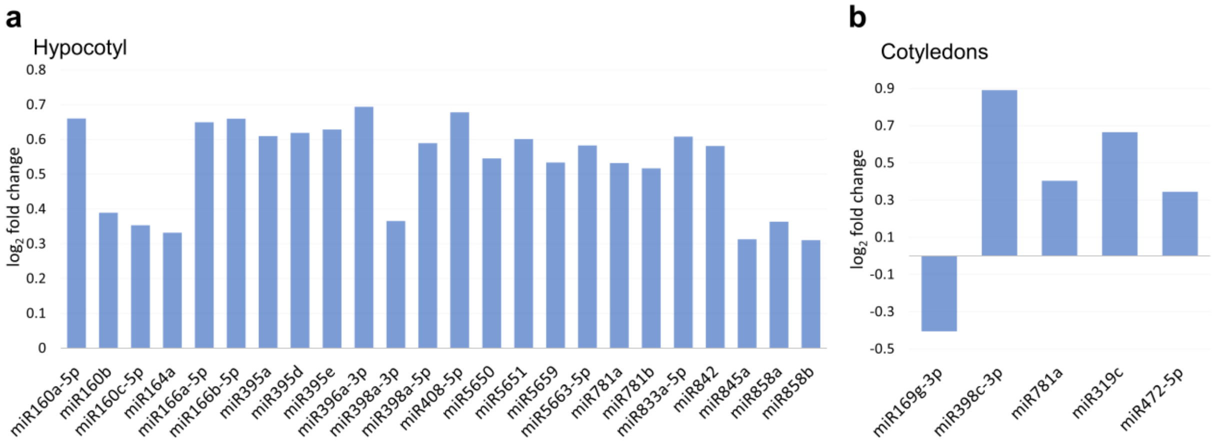

Fig. S7 Differentially expressed miRNA genes in *elo3-6* (a) hypocotyl and (b) cotyledons.

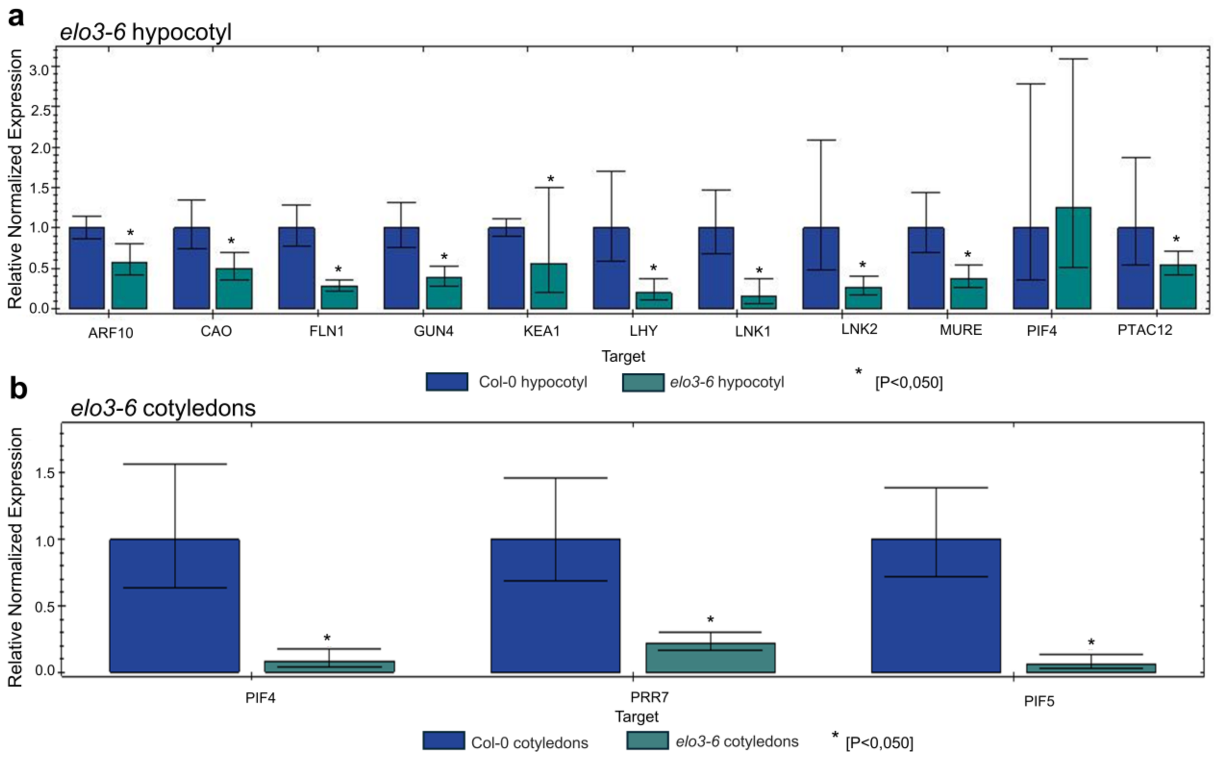

Fig. S8 Relative normalized expression of selected genes in *elo3-6* hypocotyl (a) and cotyledons (b) compared with gene expression in WT confirmed by RT-qPCR. Significant differences are marked with asterisks (P-value < 0,05). Error bars represent standard deviation.

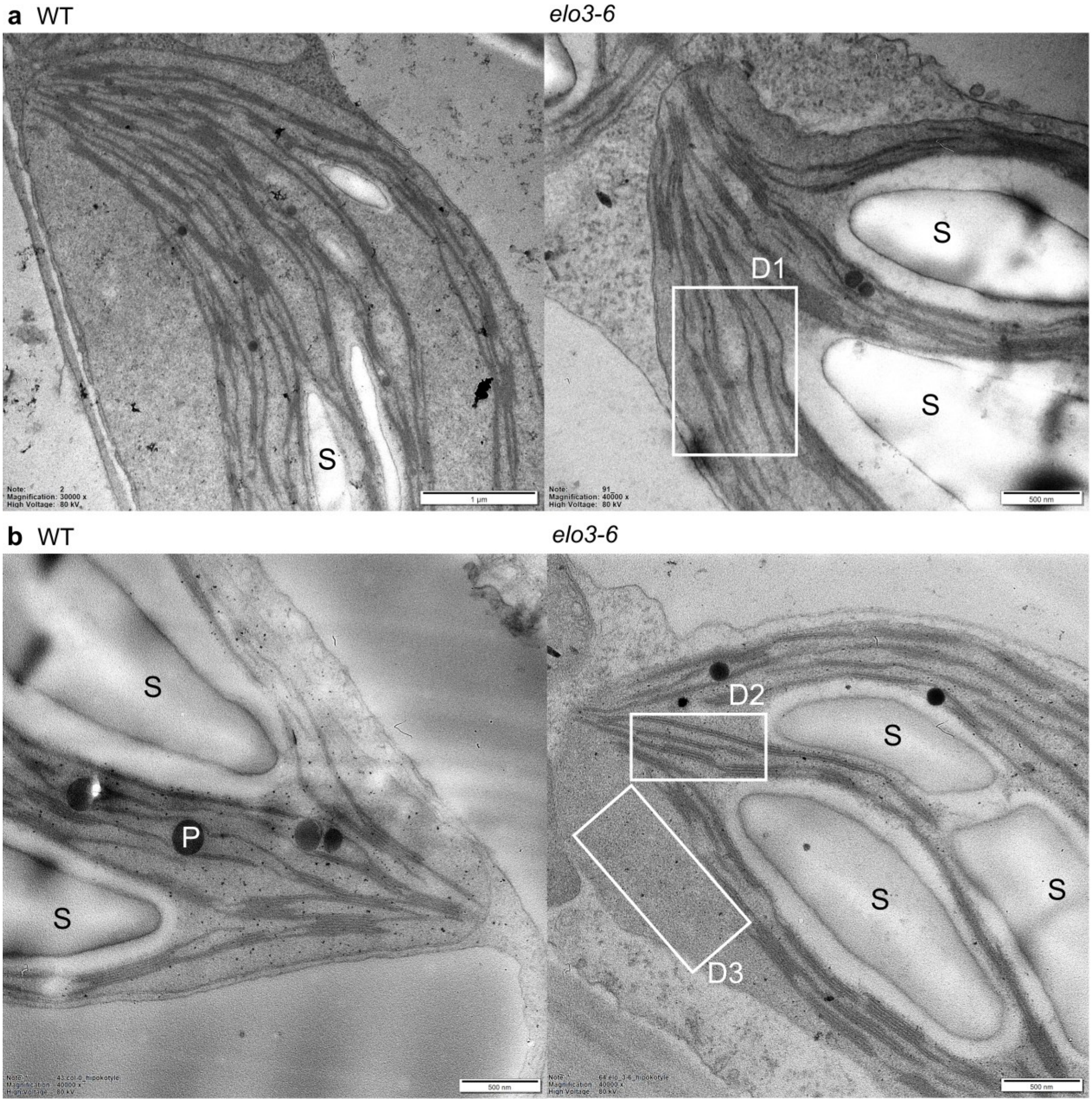

Fig. S9 Ultrastructure of chloroplasts in six-day old (a) cotyledon and (b) hypocotyl of WT and *elo3-6* mutant imaged by transmission electron microscopy. Seedlings were grown under constant white light. White bars represent scale. Most notable differences between *elo3-6* and WT are highlighted with rectangles. “P” and “S” mark plastoglobules and starch grains, respectively.

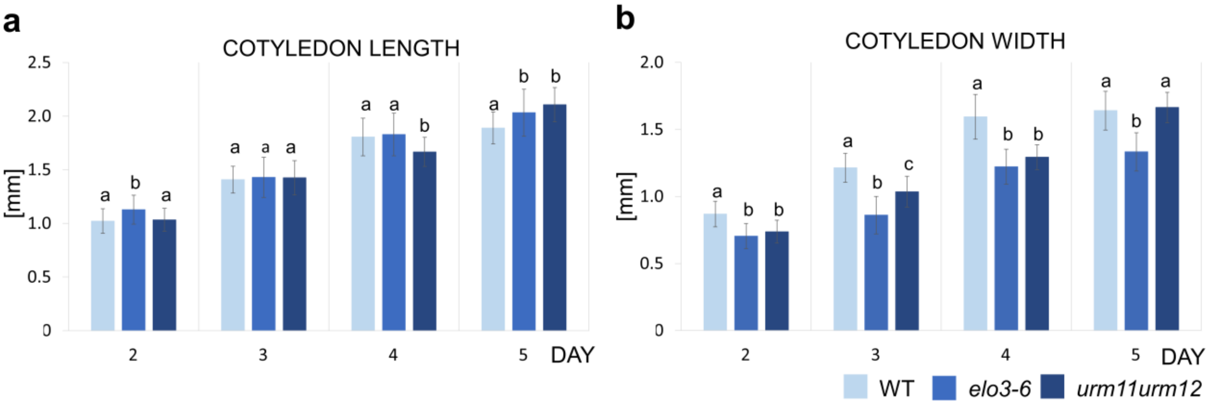

Fig. S10 Cotyledon dimensions in WT, *elo3-6*, and *urm11urm12* seedlings. Seedlings were grown under constant white light and at times indicated (a) cotyledon length and (b) width were determined. Mean values with standard deviation are shown. The letters above bars indicate groups that occurred statistically significant different.

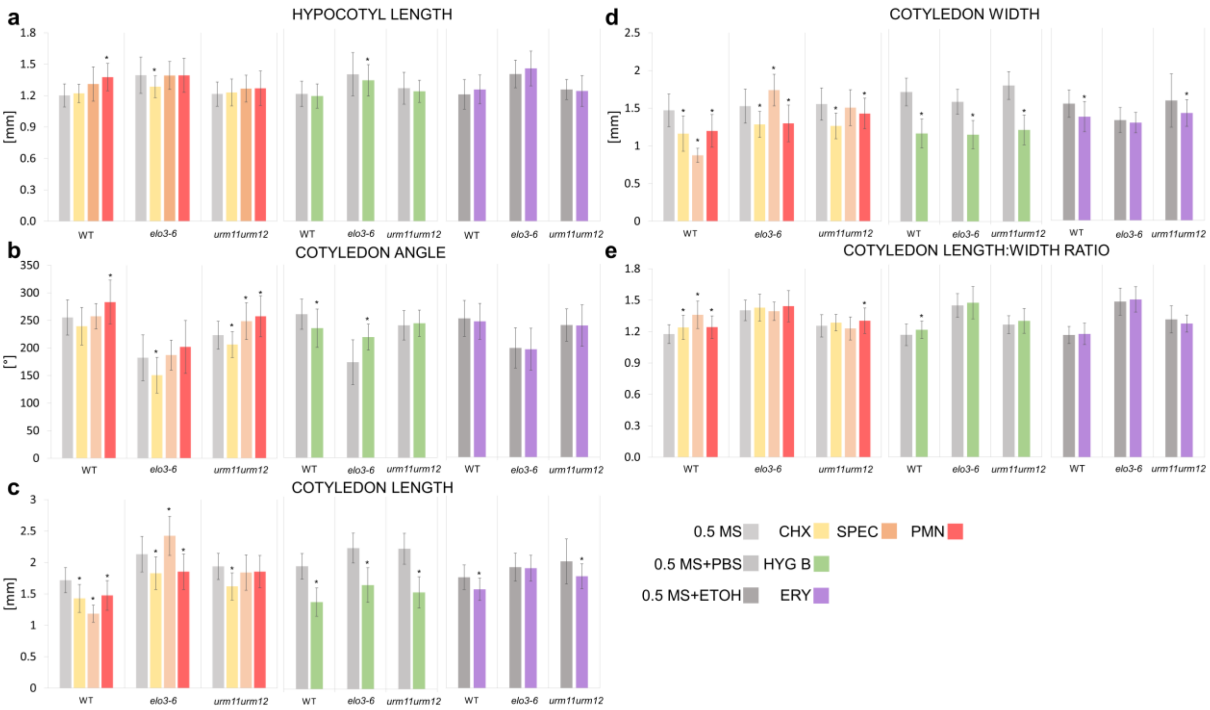

Fig. S11 Morphological traits of five-day-old WT, *elo3-6*, and *urm11urm12* seedlings. Seedlings were grown under constant white light in the absence (control) or presence of indicated translation inhibitors. (a–e) Mean values (with standard deviation) of indicated parameters. Asterisks indicate statistically significant difference between seedlings grown on translation inhibitor and control.
