# Supplementary material for: Elongator complex differentially regulates transcription and translation in the hypocotyl and cotyledons during early light-dependent Arabidopsis development
Source: Plant Cell Physiol. 2026 Jan 16;67(5):826–46. doi: 10.1093/pcp/pcag005 (PMC13227157; doi:10.1093/pcp/pcag005)
Supplement: pcp-2025-e-00159-File010_pcag005 [file pcp-2025-e-00159-file010_pcag005.pdf]

Table S1 Genes significantly downregulated in both cotyledons and hypocotyl in *elo3-6*, and differentially expressed genes with an opposite expression pattern in each organ. Only representative genes deemed likely to affect photomorphogenesis are shown.

			Hypocotyl	Cotyledons
Biological process	ID	Gene name	LFC	LFC
circadian rhythm	AT5G64170	*LNK1*	-1.27	-1.07
	AT3G54500	*LNK2*	-2.19	-0.80
	AT3G12320	*LNK3*	-2.26	-1.09
	AT5G06980	*LNK4*	-1.42	-0.76
photosynthesis	AT2G05100	*LHCB2.1*	-1.42	-0.88
	AT2G05070	*LHCB2.2*	-1.36	-1.09
	AT5G54270	*LHCB3*	-1.10	-0.71
light responses	AT1G06040	*BBX24*	-0.85	-0.86
auxin responses	AT1G04240	*IAA3*	-1.28	-0.74
circadian rhythm	AT5G24470	*APRR5*	1.35	-2.10
	AT3G46640	*LUX*	1.41	-0.76
	AT1G22770	*GI*	1.50	-2.98
light responses	AT2G31380	*BBX25*	-2.12	0.51
	AT3G15354	*SPA3*	-1.38	0.86
photosynthesis	AT1G19150	*LHCA6*	-0.72	0.56
	AT4G14690	*ELIP2*	-1.36	1.03
	AT5G13630	*GUN5*	-1.34	0.56
chloroplast organization	AT1G01790	*KEA1*	-0.70	0.59
	AT4G04850	*KEA3*	-0.62	0.68
	AT4G23940	*FTSHI1*	-1.10	0.77
	AT4G16390	*SVR7*	-1.37	0.56
	AT5G18570	*OBGL*	-1.07	0.56

Table S2 Characteristics of absolute values of $\log_2$ fold change (LFC) in various genes sets expressed in cotyledons and hypocotyl between *elo3-6* and WT.

Gene set	Cotyledons			Hypocotyl			P-value
	Number	Mean±SD	Median [Q1-Q3]	Number	Mean±SD	Median [Q1-Q3]	
all	16977	0.31±0.30	0.24 [0.11-0.42]	19473	0.45±0.44	0.31 [0.14-0.61]	1.9E-175
all with significant change	2572	0.81±0.38	0.71 [0.55-0.97]	2010	1.36±0.53	1.27 [0.99-1.59]	0
shared	16816	0.31±0.30	0.24 [0.11-0.42]	16816	0.39±0.38	0.29 [0.13-0.54]	6.2E-124
shared with significant change	471	0.99±0.48	0.88 [0.67-1.16]	471	1.31±0.56	1.18 [0.94-1.5]	5.0E-38

Q1 is quartile 1, i.e. the 25th percentile; Q3 is quartile 3, i.e. the 75th percentile.

Table S3 Differentially expressed genes in *elo3-6* hypocotyl. Only representative genes deemed likely to affect photomorphogenesis (among the downregulated genes) and hypoxia (among the upregulated genes) are shown. The expression of genes marked with an asterisk (*) was verified by RT-qPCR.

Decreased expression							
Biological Process	ID	Gene name	LFC	Biological Process	ID	Gene name	LFC
thylakoid membrane biogenesis	AT1G01790	*KEA1**	-0.70	transcription machinery	AT3G48500	*PAP3/PTAC10*	-1.22
	AT3G27750	*THA8*	-1.15		AT5G23310	*PAP4/FSD3*	-1.04
	AT5G15450	*CLB3*	-1.25		AT2G34640	*PAP5/PTAC12/HMR**	-1.10
	AT1G63680	*MURE**	-1.27		AT3G54090	*PAP6/FLN1**	-1.19
photosystems	ATCG00350	*PsaA*	-1.55		AT4G20130	*PAP7/PTAC14*	-1.31
	ATCG00340	*PsaB*	-1.20		AT3G06730	*PAP10*	-1.25
	ATCG00020	*PsbA*	-1.08		AT1G64860	*SIG1*	-0.75
	ATCG00280	*PsbC*	-1.29		AT5G24120	*SIG5*	-1.26
	AT1G19150	*LHCA6*	-0.72		AT1G09340	*CRB*	-0.73
	AT2G05100	*LHCB2.1*	-1.42		AT1G69200	*FLN2*	-1.39
	AT2G05070	*LHCB2.2*	-1.36		AT5G48470	*PRDA1*	-1.16
	AT3G27690	*LHCB2.4*	-1.56	establishment of plastid localization	AT3G45780	*PHOT1*	-0.68
	AT5G54270	*LHCB3*	-1.10		AT5G58140	*PHOT2*	-0.72
	AT3G08940	*LHCB4.2*	-1.16		AT1G75100	*JAC1*	-1.74
	AT1G55480	*MET1*	-0.57		AT1G42550	*PMI1*	-0.66
	AT4G35250	*HCF244*	-0.64	circadian clock	AT1G01060	*LHY**	-3.35
	AT1G68830	*STN7*	-0.57		AT2G46830	*CCA1*	-2.97
NAD(P)H dehydrogenase complex	AT1G15980	*PNSB1*	-0.79		AT5G64170	*LNK1**	-1.27
	AT1G64770	*PNSB2*	-0.96		AT3G54500	*LNK2**	-2.19
	AT1G55370	*NDF5*	-1.34		AT3G12320	*LNK3*	-2.26
	AT5G52100	*CRR1*	-1.05		AT5G06980	*LNK4*	-1.42
response to light	AT1G14280	*PKS2*	-1,37		AT5G17300	*RVE1*	-1.85
	AT4G18390	*TCP2*	-1,22	microtubule-based movement	AT5G51600	*MAP65-3*	-1.19
	AT1G06040	*BBX24*	-0,85		AT4G38950	*KIN7F*	-1.73
protein targeting to chloroplasts	AT4G24280	*HSP70-6*	-0.93		AT5G54670	*KIN8A*	-0.94
	AT5G57180	*CIA2*	-1.36		AT1G18550	*KIN10B*	-1.16
	AT2G47450	*CPSRP43*	-0.99		AT5G02370	*KIN14N*	-0.81
chlorophyll biosynthesis	AT1G58290	*HEMA1*	-0.98	auxin responses	AT1G04240	*IAA3*	-1.28
	AT3G48730	*GSA2*	-1.09		AT2G21050	*LAX2*	-0.95
	AT5G08280	*HEMC*	-1.04		AT1G70940	*PIN3*	-0.98
	AT2G40490	*HEME2*	-0.93		AT4G14560	*IAA1*	-1.30
	AT5G13630	*GUN5*	-1.34		AT2G22670	*IAA8*	-0.78
	AT4G18480	*CHLI1*	-0.95		AT1G04550	*IAA12*	-0.81
	AT5G45930	*CHLI2*	-1.45		AT2G28350	*ARF10**	-0.67
	AT3G59400	*GUN4**	-1.12		AT1G19220	*ARF19*	-0.81
	AT3G56940	*CRD1*	-1.21		AT5G18080	*SAUR24*	-1.38
	AT5G18660	*DVR*	-1.11		AT4G34760	*SAUR50*	-1.75
	AT1G03630	*PORC*	-1.19		AT1G29510	*SAUR67*	-1.23
	AT3G51820	*CHLG*	-0.57		AT5G54510	*GH3.6*	-0.89
	AT1G44446	*CAO**	-1.49		AT1G28130	*GH3.17*	-0.86
phytochromobilin biosynthesis	AT3G09150	*GUN3/HY2*	-1.01		AT5G55540	*LOP1*	-1
abscisic acid regulators	AT1G52400	*BGLU18*	-1.61	water homeostasis	AT2G05100	*LHCB2.1*	-1.42
	AT5G67030	*ABA1*	-1.37		AT2G05070	*LHCB2.2*	-1.36
					AT3G27690	*LHCB2.4*	-1.56
					AT1G52400	*BGLU18*	-1.61
					AT5G67030	*ZEP*	-1.37
Increased expression							
ethylene-activated signaling pathway	AT4G17500	*ERF1A*	1.86		AT1G07000	*EXO70B2*	1.18
	AT5G47220	*ERF2*	1.90		AT3G50500	*SRK2D*	0.77

	AT5G47230	*ERF5*	1.37	response to abscisic acid (stomata closure)	AT4G01026	*PYL7*	0.97
	AT3G16770	*ERF072/ RAP2.3*	2.22		AT4G26080	*ABI1*	1.07
	AT3G14230	*ERF075/ RAP2.2*	0.59	light responses	AT4G39070	*BBX20*	1.24
	AT5G25350	*EBF2*	0.80		AT1G78600	*BBX22*	1.39
	AT5G22270	*SIED1*	2.05		AT5G52250	*RUP1*	1.09
response to ROS	AT1G08830	*CSD1*	0.91		AT2G02950	*PKS1*	1.07
	AT3G01420	*DOX1*	1.34		AT4G17230	*SCL13*	1.3
	AT1G20620	*CAT3*	1.38	circadian clock	AT1G09530	*PIF3*	1.07
response to toxic substance	AT1G27130	*GSTU13*	1.04		AT3G46640	*LUX*	1.41
	AT1G17190	*GSTU26*	0.92		AT2G40080	*ELF4*	1.51
	AT2G30870	*GSTF10*	1.58		AT1G22770	*GI*	1.50
	AT1G68850	*PER11*	1.63		AT5G60100	*APRR3*	1.37
	AT2G37130	*PER21*	1.51		AT5G24470	*APRR5*	1.35
	AT3G49110	*PER33*	1.54		AT5G59570	*BOA*	1.31
	AT5G39580	*PER62*	3.11		AT1G68050	*ADO3*	2.13
	AT5G66390	*PER72*	1.4	BR homeostasis	AT5G08790	*ATAF2*	0.83
	AT2G04040	*DTX1*	1.62				
	AT4G29140	*DTX51*	1.01				

Table S4 Differentially expressed genes in *elo3-6* cotyledons. Only representative genes deemed likely to affect photomorphogenesis (among the downregulated genes) and energy metabolism and ribosome structure (among the upregulated genes) are shown. The expression of genes marked with an asterisk (*) was verified by RT-qPCR.

Biological Process	ID	Gene name	LFC	Biological Process	ID	Gene name	LFC
Decreased expression							
photosynthesis	AT3G54890	LHCA1	-0.81	circadian clock	AT5G64170	LNK1	-1.07
	AT1G61520	LHCA3	-0.59		AT3G54500	LNK2	-0.80
	AT3G47470	LHCA4	-0.79		AT3G12320	LNK3	-1.09
	AT1G29920	LHCB1.1	-0.97		AT5G06980	LNK4	-0.76
	AT1G29910	LHCB1.2	-1.00		AT5G24470	APRR5	-2.10
	AT2G34430	LHCB1.4	-0.95		AT5G02810	APRR7*	-2.77
	AT2G34420	LHCB1.5	-0.72		AT3G46640	LUX	-0.76
	AT2G05100	LHCB2.1	-0.88		AT1G22770	GI	-2.98
	AT2G05070	LHCB2.2	-1.09		AT1G18330	RVE7	-1.20
	AT5G54270	LHCB3	-0.71	light responses	AT1G78600	BBX22	-1.42
	AT2G20260	PSAE2	-0.54		AT1G09570	PHYA	-0.75
	AT1G55670	PSAG	-0.61		AT2G43010	PIF4*	-1.98
	AT5G64040	PSAN	-0.51		AT3G59060	PIF5*	-0.75
	ATCG00270	psbD	-0.52				
	ATCG01250	NDHB.2	-0.78				
	ATCG00440	NDHC	-0.72				
	ATCG00420	NDHJ	-0.76				
	ATCG00430	NDHK	-0.81				
	ATCG00480	atpB	-0.71				
	ATCG00470	atpE	-0.96				
Increased expression							
light reposnes	AT3G15354	SPA3	0.86	components of ribosomal subunits	AT2G31610	uS3z	0.52
	AT1G53090	SPA4	0.94		AT5G35530	uS3x	0.55
	AT2G31380	BBX25	0.51		AT2G37270	uS7z	0.54
Calvin-Benson cycle	AT1G67090	RBCS-1A	0.51		AT4G25740	eS10z	0.56
	AT5G38430	RBCS-1B	1.03		AT2G09990	uS9z	0.79
	AT5G38420	RBCS-2B	1.01		AT5G18380	uS9x	0.56
	AT1G56190	PGK2	0.76		AT5G61170	eS19x	0.57
	AT1G79550	PGK3	0.81		AT2G40590	eS26y	0.55
	AT3G26650	GAPA1	1.03		AT2G47110	eS31y	0.61
	AT1G42970	GAPB	0.60		AT3G09630	uL4z	0.59
	AT3G60750	TKL1	1.15		AT5G02870	uL4y	0.52
NDH complex	AT4G37925	ndhM	0.68		AT2G18020	uL2z	0.53
	AT5G58260	ndhN	0.80		AT3G27850	bL12cx	0.57
	AT1G74880	ndhO	0.72		AT4G13170	uL13x	0.51
	AT2G47910	CRR6	1.06		AT4G16720	eL15z	0.62
	AT5G39210	CRR7	0.88		AT5G27850	eL18x	0.59
	AT2G28000	CPN60A1	0.72		AT1G70600	uL15x	0.77
RuBisCO regulation	AT2G39730	RCA	0.74		AT4G15000	eL27x	0.56
	AT5G36700	PGLP1A	0.83		AT1G07070	eL33w	0.68
photorespiration	AT5G36790	PGLP1B	0.72		AT2G39390	uL29y	0.64
	AT1G68010	HPR	0.84		AT3G60245	eL43y	0.63
	AT2G35370	GDH1	0.71		AT2G36170	eL40z	0.57
	AT5G04140	GLU1	0.95		AT3G09200	uL10y	0.50
	AT3G04120	GAPC1	0.72		AT5G17870	cL38	0.63
glucose metabolism	AT1G13440	GAPC2	0.75		AT1G43170	uL3z	0.70
	AT2G21330	FBA1	0.96		AT4G05400	mL40	0.52
	AT3G52930	FBA8	0.61		AT1G57860	eL21w	0.56
	AT3G54050	CFBP1	0.60		AT4G00810	P1z	0.55
embryogenesis	AT5G63420	RNJ	0.98		AT1G18080	RACK1z	0.56
					AT1G48630	RACK1y	0.76

Table S5 Parameters of Elongator-targeted codons in differentially expressed genes (DEGs) between *elo3-6* and WT in two organs. The analysis included 555 downregulated and 893 upregulated in hypocotyl and 714 downregulated and 833 upregulated in cotyledons.

Organ	Parameter	Genes downregulated		Genes upregulated		P-value
		Mean±SD	Median [Q1-Q2]	Mean±SD	Median [Q1-Q2]	
Cotyledons	FT	0.216±0.043	0.215 [0.189-0.243]	0.206±0.04	0.209 [0.182-0.232]	4.7E-05
	FS	0.331±0.059	0.338 [0.298-0.368]	0.318±0.057	0.324 [0.288-0.355]	1.1E-05
	F2	0.039±0.014	0.038 [0.03-0.048]	0.036±0.013	0.036 [0.027-0.044]	1.2E-05
	F3	0.011±0.007	0.01 [0.006-0.014]	0.009±0.006	0.008 [0.005-0.012]	7.5E-06
	F4	0.003±0.004	0.002 [0-0.005]	0.002±0.003	0.002 [0-0.004]	4.1E-04
	D	3.686±1.021	3.538 [3.01-4.194]	3.947±1.081	3.701 [3.248-4.384]	3.3E-06
Hypocotyl	FT	0.224±0.041	0.224 [0.195-0.252]	0.205±0.044	0.205 [0.179-0.23]	1.9E-15
	FS	0.337±0.051	0.341 [0.305-0.372]	0.317±0.063	0.322 [0.281-0.355]	2.3E-10
	FT	0.041±0.014	0.041 [0.032-0.05]	0.036±0.015	0.036 [0.027-0.045]	5.1E-10
	FS	0.011±0.007	0.01 [0.006-0.015]	0.009±0.007	0.008 [0.004-0.012]	1.1E-08
	F2	0.003±0.004	0.003 [0-0.005]	0.003±0.004	0.001 [0-0.004]	8.2E-08
	F3	3.545±0.897	3.383 [2.912-4.008]	3.958±1.326	3.716 [3.217-4.418]	7.8E-12

FT is the total frequency of selected codons among all codons, FS is the relative frequency of selected codons among synonymous ones, F2, F3 and F4 are the fraction of two, three or four selected codons appearing consecutively, respectively; D is the mean distance between selected codons; Q1 is quartile 1, i.e. the 25th percentile; Q3 is quartile 3, i.e. the 75th percentile.

Table S6 The log_2 fold change (LFC) in at least 1% of genes from two organs considered separately and selected from the bottom and top of a ranking, where genes were ordered in ascending order based on codon parameters.

Organ	Parameter	Bottom of the ranking (large values of parameters)				Top of the ranking (small values of parameters)				P-value
		Parameter value	Number	Mean±SD	Median [Q1-Q2]	Parameter value	Number	Mean±SD	Median [Q1-Q2]	
Cotyledons	FT	≥ 0.31	167	-0.159±0.502	-0.165 [-0.371-0.169]	≤ 0.12	170	0.062±0.47	0.138 [-0.159-0.361]	3.1E-07
	FS	≥ 0.45	167	-0.154±0.587	-0.114 [-0.411-0.164]	≤ 0.18	167	0.091±0.484	0.191 [-0.118-0.378]	6.7E-08
	F2	≥ 0.074	167	-0.138±0.505	-0.165 [-0.365-0.145]	≤ 0.007	167	-0.014±0.437	0.04 [-0.285-0.279]	5.5E-04
	F3	≥ 0.031	167	-0.197±0.475	-0.173 [-0.469-0.098]	= 0	1361	-0.018±0.469	-0.01 [-0.259-0.241]	6.4E-07
	F4	≥ 0.015	171	-0.142±0.52	-0.161 [-0.372-0.148]	= 0	6353	-0.008±0.452	-0.011 [-0.249-0.245]	2.5E-04
	D	≥ 7.2	167	0.08±0.452	0.158 [-0.149-0.36]	≤ 2.1	167	-0.163±0.492	-0.189 [-0.401-0.159]	3.9E-08
Hypocotyl	FT	≥ 0.32	192	0.114±0.737	0.036 [-0.318-0.477]	≤ 0.11	192	0.263±0.591	0.196 [-0.103-0.598]	0.0051
	FS	≥ 0.46	192	0.143±0.745	0.119 [-0.314-0.539]	≤ 0.18	192	0.27±0.609	0.19 [-0.156-0.617]	0.0937
	F2	≥ 0.075	192	0.212±0.799	0.121 [-0.3-0.574]	≤ 0.007	193	0.282±0.534	0.236 [-0.084-0.65]	0.0943
	F3	≥ 0.032	192	0.16±0.821	0.038 [-0.321-0.562]	= 0	1607	0.219±0.684	0.166 [-0.157-0.538]	0.0327
	F4	≥ 0.015	192	0.185±0.825	0.066 [-0.29-0.552]	= 0	7338	0.127±0.64	0.063 [-0.239-0.428]	0.7279
	D	≥ 7.2	193	0.202±0.585	0.18 [-0.14-0.481]	≤ 2.0	192	0.168±0.735	0.071 [-0.286-0.569]	0.2699

FT is the total frequency of selected codons among all codons, FS is the relative frequency of selected codons among synonymous ones, F2, F3 and F4 are the fraction of two, three or four selected codons appearing consecutively, respectively; D is the mean distance between selected codons; Q1 is quartile 1, i.e. the 25th percentile; Q3 is quartile 3, i.e. the 75th percentile.

Table S7 The log_2 fold change (LFC) in at least1% of genes compared between the two organs and selected from the bottom (for FT, FS, F2, F3 and F4) and top (for D) of a ranking, where genes were ordered in ascending order based on codon parameters.

Parameter	Cotyledons			Hypocotyl			P-value
	Number	Mean±SD	Median [Q1-Q2]	Number	Mean±SD	Median [Q1-Q2]	
FT ≥ 0.32	159	-0.153±0.505	-0.157 [-0.363-0.176]	200	0.124±0.731	0.042 [-0.3-0.477]	9.6E-04
FS ≥ 0.45	154	-0.152±0.556	-0.113 [-0.408-0.167]	204	0.147±0.751	0.128 [-0.314-0.539]	1.2E-04
F2 ≥ 0.075	154	-0.141±0.504	-0.164 [-0.379-0.138]	205	0.207±0.791	0.123 [-0.294-0.543]	1.6E-05
F3 ≥ 0.031	152	-0.211±0.483	-0.196 [-0.473-0.076]	207	0.166±0.814	0.045 [-0.312-0.629]	7.8E-06
F4 ≥ 0.015	156	-0.147±0.501	-0.154 [-0.36-0.158]	202	0.206±0.817	0.124 [-0.28-0.559]	3.7E-05
D ≤ 2.1	156	-0.154±0.504	-0.184 [-0.398-0.184]	202	0.156±0.733	0.049 [-0.288-0.567]	9.5E-05

FT is the total frequency of selected codons among all codons, FS is the relative frequency of selected codons among synonymous ones, F2, F3 and F4 are the fraction of two, three or four selected codons appearing consecutively, respectively; D is the mean distance between selected codons; Q1 is quartile 1, i.e. the 25th percentile; Q3 is quartile 3, i.e. the 75th percentile.

Table S8 Gene Ontology categories for 5% of downregulated genes with statistically significant LFC ≤ -1 characterized by high fractions of Elongator-targeted codons and short distances between them. The groups were found for 154 genes with at least one of the parameters FT, FS, F2, F3 or F4 larger than the 95th percentile or parameter D smaller than the 5th percentile of the gene ranking.

Identifier	Type	Log_2-Enrichment Fold	P-Value	Subset Ratio	Description
GO:0046620	BP	5.4	5.0E-06	4.2%	regulation of organ growth
GO:0009734	BP	3.1	1.1E-05	8.4%	auxin-activated signaling pathway
GO:0046621	BP	5.8	1.8E-05	3.5%	negative regulation of organ growth
GO:2000012	BP	4.8	5.7E-05	4.2%	regulation of auxin polar transport
GO:0009628	BP	1.3	1.6E-03	22.4%	response to abiotic stimulus
GO:0007623	BP	3.0	2.0E-02	4.9%	circadian rhythm
GO:0005622	CC	0.2	3.5E-02	90.9%	intracellular anatomical structure

Table S9 Differentially expressed miRNA genes in *elo3-6* hypocotyl and cotyledons. *miRNA target genes were predicted by the miRanda algorithm.

Gene family	Mature miRNA	Mature miRNA accession	Number of target genes*
Hypocotyl			
MIR160	ath-miR160a-5p	MIMAT0000178	159
	ath-miR160b	MIMAT0000179	
	ath-miR160c-5p	MIMAT0000180	
MIR164	ath-miR164a	MIMAT0000185	1688
MIR166	ath-miR166a-5p	MIMAT0031880	584
	ath-miR166b-5p	MIMAT0031881	
MIR395	ath-miR395a	MIMAT0000938	415
	ath-miR395d	MIMAT0000941	
	ath-miR395e	MIMAT0000942	
MIR396	ath-miR396a-3p	MIMAT0031908	96
MIR398	ath-miR398a-3p	MIMAT0000948	275
	ath-miR398a-5p	MIMAT0031910	349
MIR408	ath-miR408-5p	MIMAT0031915	436
	ath-miR5650	MIMAT0022416	123
	ath-miR5651	MIMAT0022422	83
	ath-miR5659	MIMAT0022432	997
	ath-miR5663-5p	MIMAT0022440	99
MIR781	ath-miR781a	MIMAT0003940	133
	ath-miR781b	MIMAT0022420	
MIR833	ath-miR833a-5p	MIMAT0004252	365
MIR842	ath-miR842	MIMAT0004264	506
MIR845_1	ath-miR845a	MIMAT0004268	259
MIR858	ath-miR858a	MIMAT0004302	388
MIR160	ath-miR858b	MIMAT0022417	490
Cotyledons			
MIR169_2	ath-miR169g-3p	MIMAT0000912	590
MIR159	ath-miR319c	MIMAT0001016	378
MIR398	ath-miR398c-3p	MIMAT0000950	311
MIR482	ath-miR472-5p	MIMAT0032014	159
MIR781	ath-miR781a	MIMAT0003940	133

Table S10 Function and target genes of differentially expressed miRNAs associated with growth and/or chloroplast functionality in *elo3-6*.

Gene family	Target gene	miRNA Function	References
MIR160	*ARF10*, *ARF16*, *ARF17*	Promotes hypocotyl elongation in response to light via cleavage of mRNA of auxin response factors (ARFs).	(Dai et al., 2021, p. 202)
MIR166	*PHB*, *PHV*, *REV*, *ATHB8*, *ATHB15*	miR166 targets HD-ZIP III transcription factors regulating development of the shoot apical meristem and vascular tissues. *PHB*, *PHV*, and *REV* are involved in regulating dorsiventrality in leaf.	(Byrne, 2006; Li et al., 2016; Ramachandran et al., 2017)
MIR395	*GUN5*	Suppresses the expression of *GUN5*, encoding Mg-chelatase involved in chlorophyll biosynthesis and retrograde signaling, in the presence of brassinosteroids.	(Ibata et al., 2016; Lin et al., 2013)
MIR396	*GRF1-4,7-9*, *bHLH74*, *CER1*, *CER2*	Represses the expression of *GRFs*, which control cell proliferation in leaves.<br>Represses the expression of *bHLH74*, which promotes cell elongation.<br>Negatively regulates the expression of ceradmidase-like genes *CER1* and *CER2*.	(Debernardi et al., 2012; Liang et al., 2014; Liu et al., 2009)
MIR408	*LAC3*,*12*,*13*, *UCC2*, *PLC*	Under high light and copper abundance HY5 and SPL7 promote miR408 expression, which represses the expression of laccases, uclacyanin 2, and plantacyanin regulating photosynthetic electron transport.	(Gao et al., 2022)
MIR858	*MYB3*	HY5 promotes the expression of miR858, which together with miPEP858a peptide modulates *MYB3* expression. MYB3 promotes *PSK4* expression encoding regulator of auxin signaling and expansins action.	(Badola et al., 2022; Sharma et al., 2022)
MIR319	*TCP2-4*,*10*,*24 (JAW-TCPs)*	Represses the accumulation of JAW-TCPs transcripts allowing to maintain the balance between cell proliferation and differentiation during leaf morphogenesis.<br>TCP4, targeted by miR319, promotes *VND7* expression triggering secondary cell wall biosynthesis and programmed cell death.	(Shankar et al., 2023; Sun et al., 2017)

Table S11 Sequences of primes used in the RT-qPCR and PCR reactions.

RT-qPCR			
ID	Gene	Primer	Sequence
AT1G13320	*PP2A*	forward	TAACGTGGCCAAAATGATGC
		reverse	GTTCTCCACAACCGCTTGGT
AT5G64170	*LNK1*	forward	GATGTGGACAACATGCTTAGGAGT
		reverse	CTGGGCAGAAGAGAACCACC
AT3G54500	*LNK2*	forward	CTCAGTTGAGGACCAGCCATA
		reverse	GAAAGGGCCTTGCCTCAGAA
AT2G43010	*PIF4*	forward	AGGGAAACAGAAATGGAACAG
		reverse	AGCCACCTGATGAGGAACTT
AT1G01790	*KEA1*	forward	CGGAGACTCTGGAGCCTAGT
		reverse	CGCTTGCTTCACATAGCTCG
AT3G54090	*FLN1*	forward	TAGCTGTGCTTAAAGAGGCGAG
		reverse	AGCCCACCAAACTTCTTAGACC
AT1G63680	*MURE*	forward	AGAGAGGAATGTCGGGAAGC
		reverse	TGACTCTCTGGTAACCGCCA
AT3G59400	*GUN4*	forward	CGGATTTCTCTTCAATGTCAGTTCC
		reverse	CACATTGATCAACCAAGAAAGAGTC
AT2G47450	*CAO*	forward	GTAGCGGAGAGTGTGATCGG
		reverse	GACATTGTCCTGAGGCTCCC
AT1G01060	*LHY*	forward	CAGGCGTTCTTGGATTTGGAA
		reverse	TCGCCACTTACCTGTTTCGTT
AT2G34640	*PTAC12*	forward	TGACGACGAAGACGATGCAG
		reverse	TCTTTGGTTTAGGCTTGGCGG
AT3G59060	*PIF5*	forward	TACCTCACTGCAGCAGAACAGAT
		reverse	CCCATCCACATCACTTGGAGT
AT2G28350	*ARF10*	forward	ATGGCGTCTCCTTCAGGTAGCT
		reverse	GGCTGCGGAATCCTAATCTTCT
AT5G02810	*PRR7*	forward	TGCTTCCGAAAGAAGGTACGA
		reverse	CACAAATTGGCCTCGCACTC
PCR			
Genotype	Primer	Annealing temperature	Sequence
elo3-6	LP	59°C	ACCGTAAATCAGCATTTGTCG
	RP	59°C	TGGGGTTTAGGTAGTTTTGGG
	LB	59°C	ATATTGACCATCATACTCATTGC
urm11-1	LP	59°C	CCCAAATCGACAGAGAATCAG
	RP	59°C	TTTGTACGAACCCATGAAAGC
	LB	59°C	ATTTTGCCGATTTCGGAAC
urm12-2	LP	59°C	TTTAAATGATGACGCGGAAAG
	RP	59°C	TGACCACTAAGTTCCCAATCG
	LB	59°C	ATTTTGCCGATTTCGGAAC

Badola, P.K., Sharma, A., Gautam, H., and Trivedi, P.K. (2022) MicroRNA858a, its encoded peptide, and phytosulfokine regulate Arabidopsis growth and development. *Plant Physiology*. 189: 1397–1415.

Byrne, M.E. (2006) Shoot Meristem Function and Leaf Polarity: The Role of Class III HD–ZIP Genes. *PLoS Genet*. 2: e89.

Dai, X., Lu, Q., Wang, J., Wang, L., Xiang, F., and Liu, Z. (2021) MiR160 and its target genes ARF10, ARF16 and ARF17 modulate hypocotyl elongation in a light, BRZ, or PAC-dependent manner in Arabidopsis. *Plant Science*. 303: 110686.

Debernardi, J.M., Rodriguez, R.E., Mecchia, M.A., and Palatnik, J.F. (2012) Functional Specialization of the Plant miR396 Regulatory Network through Distinct MicroRNA–Target Interactions. *PLoS Genet*. 8: e1002419.

Gao, Y., Feng, B., Gao, C., Zhang, H., Wen, F., Tao, L., et al. (2022) The Evolution and Functional Roles of miR408 and Its Targets in Plants. *IJMS*. 23: 530.

Ibata, H., Nagatani, A., and Mochizuki, N. (2016) CHLH/GUN5 Function in Tetrapyrrole Metabolism Is Correlated with Plastid Signaling but not ABA Responses in Guard Cells. *Front Plant Sci*. 7.

Li, Z.-X., Li, S.-G., Zhang, L., Han, S., Li, W.-F., Xu, H., et al. (2016) Over-expression of miR166a inhibits cotyledon formation in somatic embryos and promotes lateral root development in seedlings of Larix leptolepis. *Plant Cell Tiss Organ Cult*. 127: 461–473.

Liang, G., He, H., Li, Y., Wang, F., and Yu, D. (2014) Molecular Mechanism of microRNA396 Mediating Pistil Development in Arabidopsis. *PLANT PHYSIOLOGY*. 164: 249–258.

Lin, L.-L., Wu, C.-C., Huang, H.-C., Chen, H.-J., Hsieh, H.-L., and Juan, H.-F. (2013) Identification of MicroRNA 395a in 24-Epibrassinolide-Regulated Root Growth of Arabidopsis thaliana Using MicroRNA Arrays. *IJMS*. 14: 14270–14286.

Liu, D., Song, Y., Chen, Z., and Yu, D. (2009) Ectopic expression of miR396 suppresses *GRF* target gene expression and alters leaf growth in *Arabidopsis*. *Physiologia Plantarum*. 136: 223–236.

Ramachandran, P., Carlsbecker, A., and Etchells, J.P. (2017) Class III HD-ZIPs govern vascular cell fate: an HD view on patterning and differentiation. *EXBOTJ*. 68: 55–69.

Shankar, N., Sunkara, P., and Nath, U. (2023) A double-negative feedback loop between miR319c and JAW-TCPs establishes growth pattern in incipient leaf primordia in Arabidopsis thaliana. *PLoS Genet*. 19: e1010978.

Sharma, A., Badola, P.K., Gautam, H., Gaddam, S.R., and Trivedi, P.K. (2022) HY5 regulates light-dependent expression and accumulation of miR858a-encoded peptide, miPEP858a. *Biochemical and Biophysical Research Communications*. 589: 204–208.

Sun, X., Wang, C., Xiang, N., Li, X., Yang, S., Du, J., et al. (2017) Activation of secondary cell wall biosynthesis by miR319-targeted *TCP 4* transcription factor. *Plant Biotechnology Journal*. 15: 1284–1294.
