# Supplementary material for: Elongator complex differentially regulates transcription and translation in the hypocotyl and cotyledons during early light-dependent Arabidopsis development
Source: Plant Cell Physiol. 2026 Jan 16;67(5):826–46. doi: 10.1093/pcp/pcag005 (PMC13227157; doi:10.1093/pcp/pcag005)
Supplement: pcp-2025-e-00159-File011_pcag005 [file pcp-2025-e-00159-file011_pcag005.pdf]

Methods S1 - PCR for genotyping

For genotyping, DNA was extracted from leaves using the Fast DNA Plant Screen (A&A Biotechnology) according to the manufacturer's protocol, with 50 µl of buffers A and B used. PCR was performed using PCR Mix Plus HGC (A&A Biotechnology) following to the manufacturer's protocol. The thermal cycling conditions were: initial denaturation at 95°C for 5 min; 30 cycles of 95°C for 30 s, 59°C for 30 s, and 72°C for 1 min; followed by a final extension at 72°C for 5 min. All primers are listed in Table S11.

Methods S2 - RNA-Seq

Reads classified as rRNA were removed by RiboDetector (Deng et al., 2022). Quality of non-rRNA sequencing data was checked and filtered to retain only high-quality bases by performing adapter trimming, quality filtering and per-read quality pruning. Poor quality bases were removed with fastp software (Chen et al., 2018) using the sliding window approach, taking Q20 as broader values. Adapter sequences and reads shorter than 30 bp were eliminated. Pruned reads were aligned to the *Arabidopsis* Tair10 reference genome (GCF_000001735.4) using STAR (Spliced Transcripts Alignment to a Reference), followed by further processing with the Sentieon framework. Gene quantification was performed using RSEM (Li and Dewey, 2011) based on an Expectation-Maximization (EM) algorithm. Genes receiving less than 10 reads on an average across the compared groups were removed. The abundance counts of each gene were then used to assess differential gene expression using the R/Bioconductor DESeq2 package (Love et al., 2014), which normalizes the abundance counts to account for observed variance (due to differences in sequencing depths, sample groups and replicates) generating normalized gene counts. Statistical tests were performed for each gene to compare the distributions between mutant vs. WT generating p-values for each gene. The final p-values were corrected by determining false discovery rates (FDR) using the Benjamin–Hochberg method.

Methods S3 – RT-qPCR

cDNA was synthesized using iScript cDNA Synthesis Kit for RT-qPCR (Bio-Rad) and 1 µg of total RNA. Quantitative real-time PCR was run on a Bio-Rad CFX96 Connect System instrument with dedicated software (CFX Maestro) using iTaq Universal SYBR Green Supermix (Bio-Rad) according to the manufacturer's protocol. The *PP2A* gene encoding protein phosphatase 2A was used as a reference. All primers are listed in Table S11.

Methods S4 - miRNA-Seq

Raw reads were trimmed with Cutadapt v3.0 (Martin, 2011), and checked for quality with FastQC. miRTrace tool (Kang et al., 2018) was used to characterize reads by profiling sequencing quality, read length, sequencing depth, miRNA complexity, and the proportion of miRNAs versus undesirable sequences. Bowtie (v1.3.0) (Langmead et al., 2009) was used to align reads with the *Arabidopsis* Tair10.1 (GCF_000001735.4) reference genome. Detailed analysis of miRNAs was performed with mirDeep2 (v0.1.3) (Friedländer et al., 2012), in which the reads were compared with miRbase database. Finally, differentially expressed miRNA genes were identified using the edgeR package (Robinson et al., 2010) in the R software. miRanda (Richardson et al., 2011) was used to predict sequences targeted by mature miRNAs described in the miRBase database, using the following parameters: score ≥ 150, energy ≤ -20.

Chen, S., Zhou, Y., Chen, Y., and Gu, J. (2018) fastp: an ultra-fast all-in-one FASTQ preprocessor. *Bioinformatics*. 34: i884–i890.

Deng, Z.-L., Münch, P.C., Mreches, R., and McHardy, A.C. (2022) Rapid and accurate identification of ribosomal RNA sequences via deep learning. *Nucleic Acids Res*. 50: e60–e60.

Friedländer, M.R., Mackowiak, S.D., Li, N., Chen, W., and Rajewsky, N. (2012) miRDeep2 accurately identifies known and hundreds of novel microRNA genes in seven animal clades. *Nucleic Acids Res*. 40: 37–52.

Kang, W., Eldfjell, Y., Fromm, B., Estivill, X., Biryukova, I., and Friedländer, M.R. (2018) miRTrace reveals the organismal origins of microRNA sequencing data. *Genome Biol*. 19: 213.

Langmead, B., Trapnell, C., Pop, M., and Salzberg, S.L. (2009) Ultrafast and memory-efficient alignment of short DNA sequences to the human genome. *Genome Biol*. 10: R25.

Li, B., and Dewey, C.N. (2011) RSEM: accurate transcript quantification from RNA-Seq data with or without a reference genome. *BMC Bioinformatics*. 12: 323.

Love, M.I., Huber, W., and Anders, S. (2014) Moderated estimation of fold change and dispersion for RNA-seq data with DESeq2. *Genome Biol*. 15: 550.

Martin, M. (2011) Cutadapt removes adapter sequences from high-throughput sequencing reads. *EMBnet.journal*. 17: 10.

Richardson, K., Lai, C.-Q., Parnell, L.D., Lee, Y.-C., and Ordovas, J.M. (2011) A genome-wide survey for SNPs altering microRNA seed sites identifies functional candidates in GWAS. *BMC Genomics*. 12: 504.

Robinson, M.D., McCarthy, D.J., and Smyth, G.K. (2010) edgeR : a Bioconductor package for differential expression analysis of digital gene expression data. *Bioinformatics*. 26: 139–140.
